# Effectiveness of Virtual/Augmented Reality–Based Therapeutic Interventions on Individuals With Autism Spectrum Disorder: A Comprehensive Meta-Analysis

**DOI:** 10.3389/fpsyt.2021.665326

**Published:** 2021-06-23

**Authors:** Behnam Karami, Roxana Koushki, Fariba Arabgol, Maryam Rahmani, Abdol-Hossein Vahabie

**Affiliations:** ^1^School of Cognitive Sciences, Institute for Research in Fundamental Sciences (IPM), Tehran, Iran; ^2^Cognitive Neuroscience Laboratory, German Primate Center, Goettingen, Germany; ^3^School of Medicine, Shahid Beheshti University of Medical Sciences, Tehran, Iran; ^4^Behavioral Science Research Center, Shahid Beheshti University of Medical Sciences, Tehran, Iran; ^5^Department of Psychology, Faculty of Psychology and Education, University of Tehran, Tehran, Iran; ^6^Control and Intelligent Processing Center of Excellence (CIPCE), Cognitive Systems Laboratory, School of Electrical and Computer Engineering, College of Engineering, University of Tehran, Tehran, Iran

**Keywords:** autism spectrum disorder, virtual reality, rehabilitation, technology, augmented reality

## Abstract

In recent years, the application of virtual reality (VR) for therapeutic purposes has escalated dramatically. Favorable properties of VR for engaging patients with autism, in particular, have motivated an enormous body of investigations targeting autism-related disabilities with this technology. This study aims to provide a comprehensive meta-analysis for evaluating the effectiveness of VR on the rehabilitation and training of individuals diagnosed with an autism spectrum disorder. Accordingly, we conducted a systematic search of related databases and, after screening for inclusion criteria, reviewed 33 studies for more detailed analysis. Results revealed that individuals undergoing VR training have remarkable improvements with a relatively large effect size with Hedges *g* of 0.74. Furthermore, the results of the analysis of different skills indicated diverse effectiveness. The strongest effect was observed for daily living skills (*g* = 1.15). This effect was moderate for other skills: *g* = 0.45 for cognitive skills, *g* = 0.46 for emotion regulation and recognition skills, and *g* = 0.69 for social and communication skills. Moreover, five studies that had used augmented reality also showed promising efficacy (*g* = 0.92) that calls for more research on this tool. In conclusion, the application of VR-based settings in clinical practice is highly encouraged, although their standardization and customization need more research.

## Introduction

Autism spectrum disorder (ASD) is a complex neurodevelopmental disorder characterized by impairments in social communication and social interaction in conjunction with restricted, repetitive patterns of behavior, interests, or activities (1). Affecting 1 in 68, ASD is the most prevalent psychological childhood disorder with sustained long-term effects on the quality of life of these patients (2).

Although at present there is no particular accepted treatment for ASD, there is a growing consensus that appropriately targeted individualized behavioral and educational intervention programs [e.g., Treatment and Education of Autistic and Related Communication Handicapped Children (TEACCH) program, Early Intensive Behavioral program, Applied Behavior Analytic (ABA) program, Denver model, etc.] have the potential to positively impact the lives of individuals and their families (3–7). The increasing number of individuals with ASD together with the substantial achievements that have been made thus far by this behavioral rehabilitation programs has ignited a line of research aimed at developing several technologies with the focus on improving these programs (8). Some examples include robotics (9–11), interactive video modeling (12–14), mobile and touchpad devices (15–17), wearable training systems on Google Glass (18), and virtual reality (VR) (19, 20). Interestingly, individuals with ASD have shown special interest and adherence to computerized programs (21) and learning through it (22, 23). Moreover, the burden of many hours of training by a therapist can be alleviated by using technology-based training at home.

Among these technologies, VR has become one of the most promising tools to address the psychological needs of people with ASD in various settings. Since two decades ago, VR was introduced as an effective tool in the neurocognitive rehabilitation of patients with ASD (24). This effectiveness has been approved by a decade of research afterward practicing different types of VR configurations on patients with different levels of disorder (25, 26). Besides, some efforts could have possibly improved the application of VR technology in recent works by proposing consideration of psychological theories in task design (27) and highlighting particular features of VR configurations and human–VR interactions (28). VR reduces the social pressure on the patient and provides a realistic environment for more effective training and possibly reduces the needed training hours. Current studies cover a great range of training interventions, including training of social adaptation and communication skills (29–31); emotional skills (32–34); daily living skills such as shopping (35, 36), driving (37–39), and street crossing (40, 41); and cognitive functions (42–44).

VR is a human–computer interface, which by using computer graphics generates a multidimensional environment with multiple sensory channels that allow individuals to explore the virtual environment (VE) through visual, auditory, tactile, and sometimes even olfactory perception, creating an interactive and immersive experience for the user (45, 46). VR can be implemented in head-mounted visual display (HMD) systems, head and body tracking, CAVE (Cave Automatic Virtual Environment) automatic VEs or room-like displays, and other technologies. They can be used to create a realistic sense of “presence” within a computer-generated environment (47). Augmented reality (AR), which can be considered as another type of VR, is a real-time view of an existing world that is superimposed by some virtual data. Unlike VR technology that fully submerges people in an artificial environment avoiding the existing world, AR technology enhances the feeling by overlaying the computer-generated things over the real world (48).

VR training offers several advantages; perhaps the most important one is to provide a safe access to realistic environments that would be considered dangerous in the real world along with active participation in the virtual world. Furthermore, by providing flexibility in controlling the task complexity, reinforcement through repetition and real-time visual and auditory feedback, VR enhances enjoyment and thus improves learning quality through it. These favorable properties of VR have made it a viable tool to be used in training and rehabilitation (49, 50).

In the past decade, VR has served as an effective new treatment tool in different areas such as rehabilitation in post-stroke patients (51, 52), pain management (53), phobias, posttraumatic stress disorders, obsessive–compulsive disorders, anxiety and stress disorders (54), depression (55), attention-deficit/hyperactivity disorder (ADHD) (56), cerebral palsy (57), and of course, ASD.

Although during recent years, several systematic reviews have been conducted to evaluate the efficacy of technology application on training and teaching different skills such as communication and social skills (58, 59), academic skills (60), or information processing (61), only the contribution of Mesa-Gresa et al. (62) was focused on VR and autism as an evidence-based systematic review on the effectiveness of VR-based intervention in ASD. However, their study did not provide a statistical analysis of outcomes for different clinical targets; besides, the included population in their study was limited to children and adolescents.

To date of this study, there is only one meta-analysis on technology-based intervention such as computer games, interactive DVDs, shared active surfaces, and VR in patients with autism (63). Their study presented a comprehensive meta-analysis on the technology-based intervention used in ASD people; however, the type of technology used in their included studies was mostly based on computer gaming software. Since the time of that study, the number of studies applying VR technology for training patients with autism has witnessed a dramatic surge.

Hence, we have tried to conduct a comprehensive meta-analysis focused on the effectiveness of VR technology *per se* in the training and rehabilitation of patients with autism. To achieve this goal, we performed a systematic search for studies assessing this type of intervention on the ASD population and evaluated the effectiveness of VR training on different skills including social and communication, emotion regulation, daily living, and cognitive skills (CS). We evaluated and compared the effect sizes (ESs) of different skills' improvement to appraise the most influenced clinical targets.

## Materials and Methods

### Study Identification and Selection

We systematically searched clinical and technical databases including PubMed, ERIC, Web of Science, PsycINFO, and IEEE following a comprehensive search strategy with the main search terms of *virtual reality, augmented reality, artificial reality, computer-simulated reality, virtual environment, virtual world, computer-simulated environment, mediated reality*, and *mixed reality* for intervention and the search terms of *autism spectrum disorder, pervasive children developmental disorder*, and *Asperger* for disorder, considering adjusted queries for each database. The detailed search strategy and search queries for each database can be seen in Supplementary Material. The initial search yielded a total of 1,204 articles. There was no limit on the publication date, and the search is updated until October 19, 2019.

After removing duplicate records, 915 articles remained for the preliminarily screening of titles and abstracts. Those studies presenting original work and discussed virtual or artificial realities for rehabilitation and training of the ASD population that were published in a peer-reviewed journal or peer-edited conference proceeding books were selected. Case reports, review articles, records that contained only an abstract, and records in non-English languages have been discarded. The articles left were 52 randomized clinical trials, cohort studies, and case series. The full texts of these articles were retrieved for more detailed consideration.

**Figure 1:**
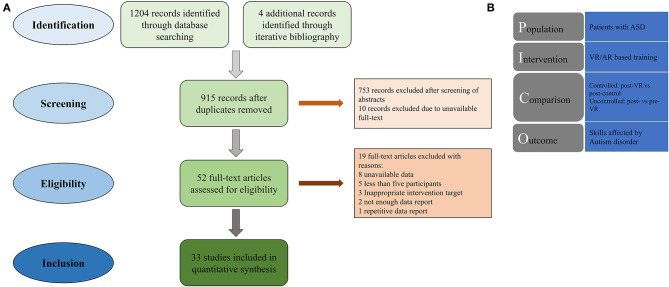
**(A)** Flow diagram of study selection and identification process. **(B)** Schematic presentation of PICO for this study.

A full-text detailed review was done using the PICO (patient, intervention, comparison, and outcome) process (Figure 1B). The criteria for including studies in our meta-analysis were (1) participants of any age were diagnosed with ASD with a formal diagnostic tool; (2) intervention was conducted on an interactive VR-based setting; (3) the designed intervention aimed at improving skills related to the core symptoms or deficits of ASD; and (4) the same measured data were available on a control group as for the intervention group that measurements performed on the intervention group before undertaking the goal intervention or on a control group that did not receive the goal intervention; and (5) intervention outcomes were assessed by a quantitative measure that was similar for the intervention and control conditions. The studies that did not comply with these criteria were excluded. Along with those, nine records that contained incomplete and/or inaccurate outcome reports in its text or figures were contacted for further information (34, 35, 64–69). One of them responded (41) and thus included in the study. Besides that, five other articles were excluded for a very small sample size (<3) (34, 70–73) and three others for an unfavorable design of the experiment (e.g., single case reports) (32, 74, 75). In the end, 33 studies were proven to be eligible for entering the meta-analysis. The flow diagram of the study selection process is presented in Figure 1A.

All the studies were coded for the following items: definite diagnosis of disease, diagnostic tool, mean and standard deviation of age of participants, number of participants assigned and completed the course of intervention in the target and control groups, contributing factors and modalities that experimenters controlled for inclusion of study population, concomitant comorbidities with ASD in participants, type of intervention technology (VR or AR), technical details of intervention, experiment properties and intensity of intervention, design of experiment (uncontrolled or case–control), purpose of experiment, and outcome measures and their descriptions.

### Coding and Defining Variables

ASD is a very heterogeneous disorder. Different patients may vary hugely in levels of deficit in different aspects of cognitive functionalities. Thus, most of the studies had attempted measuring multiple outcomes for assessment of therapeutic effectiveness. Dealing with this variability in study outcomes, we categorized them into four major categories: social and communication skills (SCS; e.g., social adaptation and interaction, communication, social reciprocity, social responsiveness, negotiation skills, theory of mind), emotion recognition and regulation skills (ERS; e.g., emotion expression, affect recognition, stress, and anxiety management), daily living skills (DLS; e.g., driving, shopping, street crossing, and job interview skills), and CS (e.g., attention and concentration, reasoning and problem solving, executive function, language, and metacognition). By this means, we were able not only to determine the general effectiveness of VR training but also to distinguish different aspects of ASD-related disabilities in terms of benefit they receive from intervention.

There was a considerable number of trials in which outcomes were assessed by a measure that was mostly intuitive and specifically designed for that experiment (e.g., number of greeting with a friend in VE or subject performance in a face detection task, driving, cross walking, shopping task, or construction play task) rather than measures that are widely used in the field [e.g., Social Responsiveness Scale (SRS) score of social awareness, PEP-3 score affective expressions, Adaptive Behavior Assessment System score of leisure, etc.]. We classified these two types of measures as non-formal and formal, respectively, and considered it as a possible moderator of measured training effectiveness for each trial. Another presumed moderator was the type of technology used for intervention, namely, VR or AR, which are characteristically different in terms of design and application. To explore the effectiveness of the intervention at any age, we assumed four age categories of 4–8, 8–12, 12–16, and older than 16 years. Each trial fell into one of these categories based on their participants' mean age. In a considerable number of trials, patients had some concomitant comorbidity along with their main disorder, ASD. To see how much this comorbidity affected the results of the intervention, we considered the presence or absence of comorbidity as another moderator and compared the results of interventions when having or not having concomitant comorbidity. These four categorical moderators were defined for further subgroup meta-analysis. The trials in which full information regarding any of these moderators was not available were excluded from analysis for that moderator.

Subgroup meta-regression was also applied to three continuous moderator variables: number of intervention sessions, sex, and publication date. These variables were defined as the number of separate sessions or visits in which intervention was applied, male ratio (number male subjects divided by the total number of subjects), and the year of publication of the study, respectively.

### Statistical Procedure

Similar to the majority of studies in the literature of training effectiveness, the pool of studies in our meta-analysis included a mixture of two major types of experiment designs, namely, controlled and uncontrolled designs. In controlled or independent-group design, one group received the training, and the other group served as a control. The difference between the groups on the outcome measure was used as an estimate of the treatment effect. On the other hand, in the uncontrolled or single-group pretest posttest design, each individual was measured before and after treatment, and the difference between the individuals' scores before and after it was used as an estimate of the treatment effect.

As the characteristic distinction between these two types of designs can lead to a significant difference in estimated ES and its precision, we opted for design-specific estimations proposed by Morris and DeShon (76) for each study. For uncontrolled studies, the repeated measure ES was calculated as the mean of change from pretest to post-test scores divided by its standard deviation, which is equivalent to the *t* statistic of paired *t*-test between two pre-test and post-test data. Then, the Standardized Mean Difference (SMD) for these trials was calculated as follows:

(1) $$\begin{array}{l} SMD\;\left(uncontrolled\right)=\frac{t}{\sqrt{n}} \end{array}$$

where *t* represents *t* statistic, and *n* represents the number of participants. We used the *t* statistic values provided in the contents of articles whenever possible or calculated them from the exact pretest and posttest scores.

For controlled studies, the SMD at the posttest was calculated as follows:

(2) $$\begin{array}{l} SMD\;\left(controlled\right)=\frac{\mu c-\mu i}{SDpool} \end{array}$$

where μ*c* represents mean of the control group, μ*i* represents mean of the intervention group, and *SDpool* is calculated as follows:

(3) $$\begin{array}{l} SDpool=\sqrt{\frac{\left(Ni-1\right)*SDi^{2}+\left(Nc-1\right)*SDc^{2}}{Ni+Nc-2}} \end{array}$$

where *Ni* is the size of the intervention group, *Nc* is the size of the control group, *SDi* is the standard deviation of the intervention group, and *SDc* is the standard deviation of the control group at posttest.

For two controlled studies in which pretest data were available for both intervention and control groups, SMD was calculated as follows:

(4) $$\begin{array}{l} SMD\;\left(prepost\;controlled\right)\\ \;=\frac{\left(\mu post_{i}-\;\mu prei\right)-\;\left(\mu postc-\mu prec\right)}{SDpre} \end{array}$$

where μ*post*_*i*_ represents the mean of intervention group scores at posttest; μ*prei*, the mean of intervention group scores at pretest; μ*postc*, the mean of control group scores at posttest; μ*prec*, the mean of control group scores at pretest; and *SDpre* is again calculated with the following equation:

(5) $$\begin{array}{l} SDpre=\sqrt{\frac{\left(Ni-1\right)*SDprei^{2}+\;\left(Nc-1\right)*SDprec^{2}}{Ni+Nc-2}} \end{array}$$

where *SDprei* and *SDprec* represent the standard deviation of the intervention and control groups' scores at the pretest, respectively (77). All of the aforementioned calculations of SMDs were done in a way that ensures the highest precision in the estimation of each experiment's ES by the available information.

The final ES indicator, Hedges *g*, then defined as the product of the output SMD and small sample correction factor $C=\frac{3}{4^{*}df+1}$ where *df* is degrees of freedom. ESs were calculated and reported so that a positive sign represents an improvement in the target skill.

After the computation of ESs for each of the trials, we found that most of them reported more than one estimated value, which is called dependent nested ESs in the literature of meta-analysis. Assuming independence between estimated values for multiple outcomes in each study is usually trivial and thus obtaining a study-level ES by averaging the values within studies might lead to some useful information loss. Handling the dependency among ES estimates, three main methods have been proposed to date: multivariate meta-analysis, three-level meta-analysis, and robust variance estimation (RVE) (78). Multivariate meta-analysis is applied when one or multiple outcomes measured in each study are from a set of known and fixed outcomes across studies. The measured outcomes in our meta-analysis were highly variable from study to study, so we could not apply multivariate analysis. Because of the small sample size of the controlled trials, some of the estimated results of the three-level analysis were underpowered and unreliable, which would question drawn conclusions based on them. So, we opted for the third introduced method, RVE. It was shown that this method accommodates well the dependence arising from multiple sources simultaneously, including multiple measures and multiple treatment groups (78) and thus can be a felicitous choice for our study. Further details on the application of the RVE method on our data are described in *Results*.

According to the guidelines of Cohen (79), an absolute ES of 0.2–0.3 is regarded as a small effect, ~0.5 as a medium effect, and from 0.8 on as a large effect.

Heterogeneity was assessed by Cochran *Q, I*^2^, and τ^2^ statistics. *I*^2^ describes the percentage of variation in studies. The smaller the *I*^2^, the lower the level of heterogeneity among estimated values. τ^2^ statistic is also a measure of between-study variance of ESs. When *Q* statistic is very small, the estimated *I*^2^ is not accurate in capturing the real heterogeneity (80). In these cases, τ^2^ is more informative specifically when comparing among subgroups with low heterogeneity.

Publication bias was investigated by visual inspection of funnel plots looking for any clue of asymmetry plus Egger intercept test (81) to validate the conclusions.

All the analyses in the main text were done using customized scripts in MathWorks' MATLAB. Three-level meta-analysis was performed in R using an available R package (82).

## Results

### Description of Studies

Thirty-three studies complied with the inclusion and exclusion criteria (see *Methods*) and entered into the meta-analysis.

The interventions were applied by a controlled experiment design in seven studies and by an uncontrolled design in 24 studies. There were two studies that recruited both types of controlled and uncontrolled designs (mixed-design) (83, 84). As these mixed-design studies included different participants in each design group, we treated them as separate uncorrelated trials. Doing so, we based our analysis on 35 independent trials obtained from 33 studies (The term *trial* refers to an independent design group consistently thereafter in this article). All in all, 540 ASD participants were included in this study, of which 360 belong to uncontrolled and 180 to controlled trials. There were also 156 ASD patients in the control arm of controlled trials who received neither VR-based nor conventional intervention.

In four controlled trials, the same outcomes were measured before (baseline) and after training in both the control and intervention arms. In the remaining five controlled trials, these measurements were done only after training, and there were no baseline data provided in any control or intervention group. Of 26 uncontrolled trials, three of them applied ABC measurement strategy in a way that outcomes were measured in three temporal phases: after the first session (pre), after the last session (post), and a while after completion of intervention (follow-up) (29, 44, 85). In the other trials, the measurements were performed before (pre) and after (post) interventions. In two trials (one from control and the other one from uncontrolled trials), measurement once was done after a non-VR conventional training, and it was repeated after VR-based target training (43, 86). The data in the first condition were labeled pre-intervention, and in the latter labeled post-intervention. A prior exposure to any type of training was neither recognized nor mentioned in the other studies. The identified pre-intervention and post-intervention data for each trial were used in computing ES statistics (see *Methods* for more detail).

The diagnostic tools used to integrate patients into the study were different across trials. For instance, in two trials, diagnosis was confirmed by the *Diagnostic and Statistical Manual of Mental Disorders, Fourth Edition* (*DSM-IV*) (83, 85), in four other trials by Autism Diagnostic Observation Schedule (ADOS) (39, 42, 87, 88), in one of them by Social Communication Questionnaire (SCQ) (89), in the other two trials by SRS-II (90, 91), in the other 6 by *DSM-V* (41, 92–94), and in another one by Childhood Autism Rating Scale (CARS) (40). The diagnostic tool in the remaining 20 trials was not mentioned. Regarding stage/level of disease, three trials included patients with high-functioning ASD (HFASD) (90, 94, 95), one trial had patients with low-functioning ASD (LFASD) (92), two trials included Asperger or pervasive developmental disorder—not otherwise specified patients (31, 87), two other trials had patients with either HFASD or Asperger (96, 97), and one trial had patients with Asperger who received the intervention (98). The level of disease in the other 26 trials was not specified. Several trials considered controlling some contributing factors in the population of their study that could potentially impact the outcome of the intervention. For example, eight trials controlled the participants for IQ score (30, 41, 42, 85, 88, 94, 95, 99), two trials for SCQ score (44, 83), one trial for SRS-II score (39), one other trial for PEP-3 score of language and motor skills (92), and one trial for ASI score (86). In six uncontrolled and three controlled trials, patients had another concomitant comorbidity or disorder alongside their main disorder, ASD. Examples include some trials that had patients with a diagnosis of ADHD (42, 44), some other trials that included patients with phobia (83, 89), and finally other trials in which some patients had an intellectual disability or language disorder (40, 41, 86, 92, 93).

AR and VR were integrated into training paradigms through various tools and platforms in the 35 identified trials. From these trials, five opted for AR and 30 for VR to deliver their intervention. They implemented most of these AR-based programs through smartphone, tablet, or desktop applications and platforms to augment three-dimensional (3D) visual features to the more simplistic features conventionally used in the training paradigms, making them more appealing and engaging for children with ASD (29, 43, 85, 98). There was one trial that used Google smart glasses equipped with blink sensors, gyroscope, camera, and display screen (44). In this trial, positive feedback was displayed on the screen whenever the participant could successfully gaze at the instructor's face and detect his/her emotion. Alternatively, most of the VR-based interventions were designed on immersive 3D VE settings in which audiovisual scenes were presented on the walls and ceiling of a room where the participant could fit in different characters in realistic social scenarios. There were also trials that training was based on a particular virtual agent that the participant could play and interact with it. Some VR interventions were also planned on desktop computers using commercial VR software and some of them on HMD devices, which are recently being more and more available and gaining popularity.

More details of included studies for uncontrolled and controlled trials are provided in Tables 1, 2, respectively.

**Table 1:** Characteristics of included studies, uncontrolled trials.

**References**	**Age**	***N***	**Type**	**Methodic details**	**Application details**	**Study purpose**	**Outcome measure**				
	**Mean (SD)**						**Name**	**Details**	**Skill**	***g***	**SE*_***g***_***
Bai et al. (98)	6.8	12	AR	Playing with augmented toys in mirror AR display	N/S	Improve and learn pretend play and representation of pretense	Pretend play frequency	Play Observation Scale	CS	0.7	0.42
							Pretend play duration			0.94	0.47
							Constructive play frequency			1.42	0.59
							Constructive play duration			1.01	0.49
									**Overall**	**1.02**	**0.6**
Bernardini et al. (100)	N/M	19	VR	Playing game with VA	Several 10- to 20-min sessions in a week for 8 weeks	Help children acquire social communication skills	Response to social partner	SAP to assess socioemotional abilities of autistics	SCS	0.07	0.42
							Initiation to social partner			0.02	0.42
							Social behavior			0.02	0.42
							Sequences of social behaviors			−0.25	0.43
							Speech toward social partner			0.06	0.42
							Missed opportunities			0.81	0.54
									**Overall**	**0.12**	**0.55**
Chen et al. (85)	11.5	6	AR	ARVMS	Seven sessions	Facial expressions and emotions of others in social situations	Performance	Instructor assessment	ERS	4.81	2.94
									**Overall**	**4.81**	**2.96**
Didehbani et al. (42)	11.4 (2.7)	30	VR	Social scenarios in customized Second Life™ VE	Ten 1-h sessions	Enhance emotion recognition, social attribution, attention and executive function	NEPSY-2 affect recognition	Facial affect recognition	ERS	0.66	0.23
							EKMAN 60	Recognition of basic emotions		0.46	0.31
							Triangle total	Understanding of social intentionality	SCS	0.38	0.22
							Triangle intentionality			0.45	0.23
							Fluid reasoning	Selective attention and concentration	CS	0.52	0.27
									**Overall**	**0.44**	**0.41**
Ip et al. (99)	8.7	33	VR	School-related social scenarios in four-sided CAVE	28 sessions	Enhance social skills and coping skills while avoid unnecessary embarrassment	Eyes test	Emotion recognition	ERS	0.53	0.29
							Affective expression			0.68	0.31
							Social reciprocity	Social reciprocity	SCS	0.6	0.3
							PEP-3 overall	Social functioning and communication		0.76	0.32
									**Overall**	**0.64**	**0.45**
Josman et al. (40)	13.2 (3)	6	VR	Street crossing in VE computer program	Eight 10- to 30-min sessions	Teach street crossing skill	N of left looking at first crosswalk	Participant performance in VR software	DLS	1.72	1.16
							N of right looking at first crosswalk			0.58	0.63
							N of left looking at second crosswalk			0	0.53
							N of right looking at second crosswalk			0.37	0.57
							total N of left looking crossing the road			1.4	0.99
							total N of right looking crossing the road			0.19	0.54
							N looked left at crosswalk with traffic light			0.18	0.54
							N looked right at crosswalk with traffic light			0.45	0.59
							N of accidents at the crosswalk with traffic light			0.75	0.69
									**Overall**	**0.63**	**0.8**
Kandalaft et al. (87)	21.2 (2.7)	8	VR	Interacting with VA in second Life^TM^ software	10 sessions	Enhancing social skills, social cognition, and social functioning	SP-total	Verbal and non-verbal emotion recognition by ACS-SP	ERS	0.89	0.56
							SP-affect			0.39	0.45
							SP-prosody			1.03	0.59
							SP-pair			0.59	0.48
							EKMAN 60	Theory of mind (ToM)	SCS	1.25	0.66
							Triangle			1.08	0.61
							SSPA	Conversation skills		0.32	0.44
									**Overall**	**0.79**	**0.64**
Ke et al. (95)	N/M	8	VR	3D virtual world designed by OpenSimulator	Average of 20.22 h, over 16–31 sessions	Enhance social skills	Responding	Performance evaluated by instructors	SCS	0.02	0.42
							Initiation			1.26	0.67
							Negotiation			1.61	0.78
							Self-identification			0.83	0.54
							Cognitive flexibility			2.09	0.96
									**Overall**	**1.16**	**0.77**
Kurniawan et al. (29)	N/M	12	AR	PECS-AR	N/S	communication ability	Communication ability score	Teacher's assessment	SCS	1.26	0.47
									**Overall**	**1.26**	**0.58**
Lamash et al. (36)	14.6 (1.8)	33	VR	Shopping training in VAP-S software	Five sessions	Shopping skills, executive cognitive and metacognitive skills	WebNeuro attention component	Evaluation of cognitive and meta-cognitive functis	CS	0.58	0.2
							WebNeuro executive function component			0.58	0.2
							WebNeuro verbal component			−0.38	0.19
							TOGGS accuracy	TOGGS, performance in shopping	DLS	1.5	0.27
							TOGGS time			0.62	0.2
							TOGGS redundancy			0.93	0.22
							TOGGS strategy usage			1.85	0.31
									**Overall**	**0.81**	**0.4**
Manju et al. (30)	4.6 (0.9)	5	VR	VE with scenes presented on wall	N/S	Social skills and attention	Likert score	Attention grasping	CS	2.39	2.07
							Likert score	Social interaction	SCS	1.6	1.47
									**Overall**	**1.99**	**1.82**
Maskey et al. (89)	11.2 (2)	9	VR	Exposure to fearful stimuli in VE	Four 20- to 30-min sessions	Reduction or treating specific phobia	SCAS-P	Children's Anxiety Scale parent score	ES	0.62	0.46
							SCAS-C	Children's Anxiety Scale child score		0.66	0.45
									**Overall**	**0.64**	**0.56**
Maskey et al. (83)	29.8	8	VR	Blue VR room	Four 20-min sessions of graded exposure	Treating phobia and anxiety	Anxiety BAI score	BAI	DLS	0.03	0.42
							Anxiety GAD score	GAD-7		−0.04	0.42
							Depression score	PHQ-9		0.32	0.44
							Quality of life (QoL) physical	WHOQOL-BREF questionnaire		−0.47	0.46
							QoL psychological	Addresses QoL		−0.03	0.42
							QoL social			−1.2	0.66
							QoL environmental			0.2	0.42
									**Overall**	**−0.17**	**0.58**
Miller et al. (101)	5.2	5	VR	HMD, Google cardboard	One session per week for 3 weeks	Improve air travel skills	Parent score	5-Point Likert score	DLS	0.98	1.03
							Researcher score			1.1	1.11
									**Overall**	**1.04**	**1.12**
Milne et al. (96)	10.5	14	VR	Interacting with VA	N/S	Teaching social skills and how to cope with bullying	Conservation skills	Performance in social scenarios measured by evaluators scoring	DLS	0.67	0.33
							Dealing with bully skills			1.09	0.39
									**Overall**	**0.88**	**0.49**
Nubia et al. (43)	6	5	AR	Pictogram recognition task	N/S	Improve attention process and appearance of verbal language	Attention process	No. of children successfully finished the attention task	CS	0.53	0.3
							Emergence of language			0.55	0.31
									**Overall**	**0.54**	**0.45**
Ross et al. (37)	18	46	VR	Driving simulation in VE	8–12 sessions	Improve attitude toward driving	DAS-PR positive attitude	Driving Attitude Scale–Parent Report	DLS	1.74	0.25
							DAS-PR negative attitude			1.07	0.19
									**Overall**	**1.41**	**0.4**
Saiano et al. (41)	24 (10)	6	VR	Street crossing and path following in VE representing a city	Ten 45-min sessions	Teaching of street crossing and path following skills	Caregiver score	Likert score questionnaire	DLS	1.85	1.23
							Parent score			0.92	0.76
							Speed	Subject performance in city surveying and street crossing		1.71	1.15
							Composite index			0.45	0.59
							Figural distance			0.75	0.69
							Path length taken			0.48	0.6
									**Overall**	**1.03**	**0.94**
Simoes et al. (93)	18.8 (4.5)	6	VR	Street crossing and bus taking in VE presented by HMD	Three 20 to 40-min sessions	Teaching bus-taking routines and effectively using bus for transformation	Action accuracy	Performance in bus taking	DLS	1.1	0.5
							Debriefing accuracy			1.8	0.69
							Global EDA	Stress level	ERS	0.66	0.66
							Bus EDA			0.81	0.72
							Streets EDA			0.51	0.61
									**Overall**	**0.98**	**0.72**
Stichter et al. (88)	12.6 (0.7)	11	VR	Social competence tasks in computer-generated 3D VE	31 sessions over a 4-month period	Enhance social competence	SRS total parent score	Social Responsiveness Scale	SCS	1.04	0.46
							SRS social awareness parent score			0.47	0.36
							SRS social cognition parent score			1.15	0.48
							SRS social communication parent score			1.26	0.51
							SRS social motivation parent score			0.75	0.41
							SRS total teacher score			0.53	0.37
							SRS social awareness teacher score			0.34	0.35
							SRS social cognition teacher score			−0.12	0.34
							SRS social communication teacher score			0.6	0.38
							SRS social motivation teacher score			0.34	0.35
							BRIEF global executive parent score	Behavior Rating Inventory of Executive Function	CS	0.68	0.39
							BRIEF behavioral regulation parent score			0.45	0.36
							BRIEF metacognition parent score			0.64	0.39
							BRIEF global executive teacher score			0.5	0.37
							BRIEF behavioral regulation teacher score			0.14	0.34
							BRIEF metacognition teacher score			0.33	0.35
							Reading in mind's eye	Student performance	ERS	0.17	0.34
							Faux pas stories			−0.35	0.35
							Strange stories			0.25	0.35
							DANVA	Child facial expression analysis		0.44	0.36
							Trail making: number letter switching	D-KEFS Delis–Kaplan executive functioning system	CS	0.17	0.34
							Design fluency: switching designs			0.62	0.38
							Design fluency: total correct designs			1.06	0.46
							Color–word interface: inhibition task			−0.03	0.34
							Color–word interface: inhibit/switch			0.16	0.34
							CPT-2 overall omission errors	Continuous performance test-II (CPT-II)		0.09	0.34
							CPT-2 overall commission errors			0.15	0.34
									**Overall**	**0.44**	**0.5**
Vahabzade et al. (44)	15 (3.4)	8	AR	Maintain gaze toward faces by AR smart glasses	One session	Improving gaze duration to faces and reducing ADHD symptoms	ABC-H score	Measure of ADHD symptoms	CS	0.72	0.51
									**Overall**	**0.72**	**0.61**
Wade et al. (38)	15.9 (1.3)	6	VR	Driving simulation in VE	Six visits of three driving sessions in 24 trials	Improve safe driving skills	Performance-based failures	Subject's performance	DLS	1.98	1.3
									**Overall**	1.98	1.34
Wade et al. (39)	15.3 (1.6)	8	VR	3D game driving simulator	Six 75-min sessions	Enhancing driving skills	Duration time	Performance	DLS	0.73	0.51
							No. of failures			1.27	0.67
									**Overall**	**1**	**0.7**
Yang et al. (94)	22.5 (3.9)	17	VR	VR-SCT computer program	Ten 1-h sessions	Emotion recognition training and ToM or sociocognitive skills improvement	ACS-SP emotion recognition	Social Perception	ERS	0.89	0.56
							ToM triangle test	ToM	SCS	1.08	0.61
									**Overall**	**0.99**	**0.67**
Yuan et al. (84)	9 (1.1)	36	VR	Social scenarios in four-sided CAVE	One 1-h session	Train emotional and social skills	PEP-3 affective expressions	Emotion expression and regulation	ERS	0.35	0.18
							PEP-3 social reciprocity	Social interaction and adaptation	SCS	0.64	0.19
									**Overall**	**0.5**	**0.38**
Zhao et al. (31)	12.4 (2.6)	12	VR	Social games in CVE	N/S	Motor skill and social interaction simultaneously	Completed pieces(/min) study 1	Performance in puzzle game	SCS	1.07	0.83
							Cooperative efficacy % study 1			0.76	0.7
							Total play time (s) study 1			0.9	0.75
							Word count of ASD subjects(/min) study 1			0.34	0.57
							Back-and-forth sentences(/min) study 1			−0.65	0.66
							Aggregate score study 1			0.76	0.7
									**Overall**	**0.53**	**0.8**

*ABC-H, Aberrant Behavioral Checklist; ACS, Advanced Clinical Solutions; ADHD, attention-deficit/hyperactivity disorder; AR, augmented reality; ARVMS, Augmented Reality Video Modeling System; BAI, Beck Anxiety Inventory; BRIEF, Behavior Rating Inventory of Executive Function; CAVE, Cave Automatic Virtual Environment; CPT, continuous performance test; CS, cognitive skills; CVE, collaborative virtual environment; DANVA, Diagnostic Analysis of Non-verbal Accuracy; DAS-PR, Driving Attitude Scale–Parent Report; DLS, daily living skills; EDA, electrodermal activity; ERS, emotion regulation and recognition skills; GAD-7, Generalized Anxiety Disorder-7; N, number of participants; N/S, not specified; NEPSY, a developmental NEuroPSYchological assessment; NM, not mentioned; PECS, picture exchange communication system; PEP, psychoeducational profile; PHQ-9, Patient Health Questionnaire-9; SAP, SCERTS assessment protocol; SCAS_C, Spence Children's Anxiety Scale–Child Version; SCAS_P, Spence Children's Anxiety Scale–Parent Version; SCQ, Social Communication Questionnaire; SCS, social and communication skills; SRS, Social Responsiveness Scale; SSPA, Social Skills Performance Assessment; TOGGS, Test of Grocery Shopping Skills; VA, virtual avatar; VE, virtual environment; VR, virtual reality; VR-SCT, virtual reality social cognition training. The bold values represent overall effectiveness for each study*.

**Table 2:** Characteristics of included studies, controlled trials.

**References**	**Age**	**Number**		**Methodic details**	**Application details**	**Study purpose**	**Outcome measure**				
	**Mean (SD)**	**NI**	**NC**				**Name**	**Details**	**Skill**	***g***	**SE*g***
Chen et al. (92)	4.9 (1.1)	11	11	3D virtual punctuation tutor	Three sessions	Improve speech	Consonants	Rated with linguistis	CS	0.36	0.45
							Vowels			0.38	0.45
										**0.37**	**0.5**
Ip et al. (33)	13.55	36	36	Social scenarios in half-CAVE	28, 30-min sessions	Improving emotion recognition, emotion expression and social reciprocity, social adaptive skills	Faces test	Emotion recognition	ERS	0.26	0.24
							Eyes test			0.14	0.24
							PEP-3 affective expressions	Emotion expression, regulation, and social reciprocity		0.44	0.24
							PEP-3 social reciprocity			0.47	0.24
							ABAS communication	Social adaptive skills	SCS	0.13	0.24
							ABAS community use			−0.64	0.25
							ABAS leisure			−0.24	0.24
							ABAS self-direction			−0.48	0.24
							ABAS social			−0.23	0.24
										**−0.05**	**0.34**
Lamash et al. (36)	14.58 (1.77)	33	23	Shopping training in VAP-S software	Five sessions	Improving shopping skills	TOGGS accuracy	Performance in shopping	DLS	1.02	0.29
										**1.02**	**0.38**
Maskey et al. (83)	10.8 (2)	16	16	Blue room VR	Four sessions	Reduce phobia in ASD patients with anxiety disorder	Target behavior rating	Rating of specific phobia change	ERS	1	0.39
							Total fearfulness	FSSC-R		−0.2	0.37
							Intense fears			−0.29	0.37
							Total anxiety score, parent	SCAS-P		0.21	0.37
							Total anxiety score, child	SCAS-C		−0.04	0.37
							Formal activity, diversity	CAPE: participation in a range of solitary and group voluntary activities		−0.14	0.37
							Formal activity, intensity			−0.1	0.37
							Informal activity, diversity			−0.24	0.37
							Informal activity, intensity			−0.28	0.37
										**0**	**0.44**
Smith et al. (91)	24.9 (6.7)	16	10	Being interviewed by VA in VR-JIT computer software	10 h	Improving job interviewing and vocational skills	Role-play performance total score	Standardized role-plays	SCS	0.52	0.43
							Job interview content score			0.39	0.43
							Hard worker			0.58	0.43
							Easy to work with/teamwork			0.32	0.42
							Sounding professional			0.25	0.42
							Negotiation skills			0.32	0.42
							Interviewee performance score	Training Experience Questionnaire by interviewee		0.49	0.43
							Sharing things positively			0.73	0.44
							Sounding honest			0	0.42
							Sounding interested in job			0.26	0.42
							Comfort level			0.46	0.43
							Establishing overall rapport			0.35	0.42
							Job interview self-confidence rating	Self-confidence measure		0.61	0.43
										**0.4**	**0.48**
Smith et al. (90)	25 (6.9)	15	8	Being interviewed by VA in VR-JIT computer software	N/S	Improving job interviewing skills	Likert score	Self-confidence	SCS	0.82	0.48
							Weeks looking for a job			0.23	0.46
							Completed interviews			0.08	0.46
										**0.37**	**0.52**
Strickland et al. (97)	18.21 (1.03)	11	11	Being interviewed by VC in JobTIPS computer program	One session	Enhancing job finding skills	Response content scale	Content of the participant's responses	DLS	2.81	0.68
							Response delivery scale	Behaviors related to greetings and farewells		0.81	0.47
										**1.8**	**0.63**
Yuan et al. (84)	8.97 (1.1)	36	36	Social scenarios in four-sided CAVE	1,1-h session	Enhancing emotional and social skills	PEP-3 affective expressions	Emotion expression and regulation	ERS	0.54	0.24
							PEP-3 social reciprocity	Social interaction and adaptation	SCS	0.66	0.25
										**0.6**	**0.34**
Zhang et al. (86)	4 (1.21)	6	5	Quiver Vision augmented reality android app	20 weeks, two 15-min sessions per week	Enhance social skills	Social score	ASI disorder score	SCS	0.14	0.69
							Communication and language			0.14	0.69
							Anticipation and flexibility			−0.28	0.69
							Symbolization			−0.2	0.69
										**−0.05**	**0.73**

*ABAS, Adaptive Behavior Assessment System; CAVE, Cave Automatic Virtual Environment; CS, cognitive skills; DLS, daily living skills; ERS, emotion regulation and recognition skills; FSSC-R, Fear Survey Schedule for Children—Revised; NC, number of participants in control group; NI, number of participants in intervention group; PEP, psychoeducational profile; SCAS_C, Spence Children's Anxiety Scale–Child Version; SCAS_P, Spence Children's Anxiety Scale–Parent Version; SCS, social and communication skills; TOGGS, Test of Grocery Shopping Skills; VA, virtual avatar; VAP-S, virtual action planning supermarket; VC, virtual character; VR-JIT, Virtual Reality Job Interview Training. The bold values represent overall effectiveness for each study*.

### Meta-Analysis

In the 35 trials entailed in our meta-analysis, ES Hedges *g* were computed for 167 total number of outcome measures, in which 45 pertained to controlled and 122 pertained to uncontrolled trials. As ESs in two groups were significantly different from each other (*p* = 0.0003, unpaired *t*-test), we were not allowed to combine them into one group, and so we have done all further analyses separately for each of them.

To compute the study-level ESs, its variance, and also between-study variance, we followed the procedure described by Hedges et al. (102). Based on this procedure, an estimate of within-study correlation (ρ) is needed to compute other statistics. As this estimate could not be extracted from most of our included studies, we ran a sensitivity analysis by choosing various values for ρ ranging from 0 to 1 and then computed other statistics based on the chosen value. The results of sensitivity analysis are given in Supplementary Table 1. By this analysis, we inferred that the value study-level computed ES estimates are sensitive to the choice of ρ, but it was an ascending function of ρ (the larger the ρ, the larger the estimated study-level ESs). Therefore, to avoid any overestimation in computing summary ESs, we fixed the value of ρ at 0 and performed the analysis with this value. It is noteworthy to say that the procedure that we applied in this study is the most parsimonious one avoiding any overstatement of results, but in a realistic situation, the observed effectiveness might be larger as assuming the existence of some level of correlation between study outcomes seems to be a rational assumption. For each trial, we first computed study-level ESs for all of the outcomes irrespective of their category applying RVE procedure, which gave us overall ES of each study. Then, we repeated this procedure on estimated ESs of outcomes in each category of each study to obtain category-based ESs of that study.

### Overall Effectiveness of VR Training

**Figure 2:**
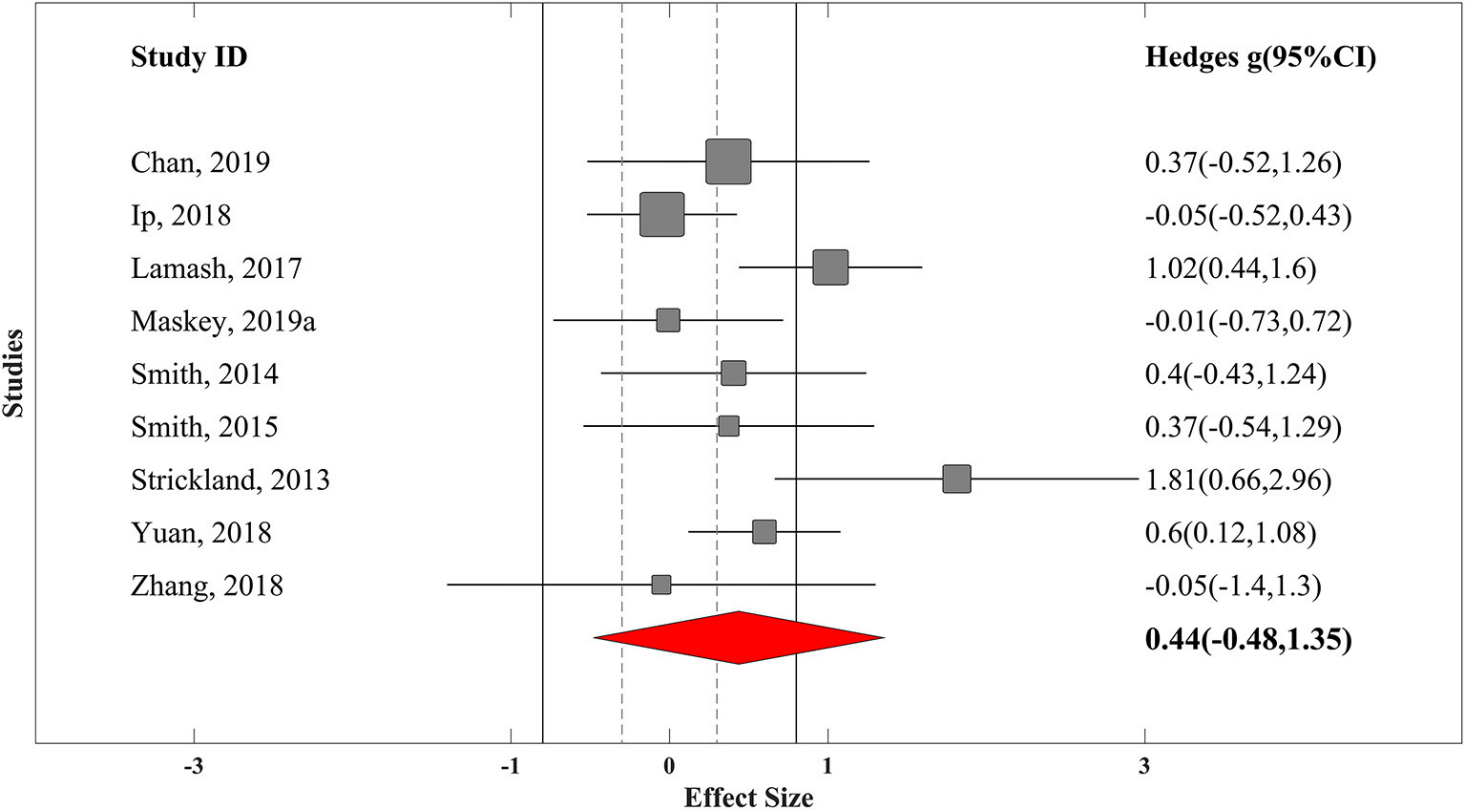
Forest plot of overall effectiveness of VR training for controlled trials with 95% confidence interval. Solid vertical lines represent strong effect size boundary (*g* = −0.8 and 0.8), and dashed vertical lines represent weak effect size boundary (*g* = −0.3 and 0.3).

**Figure 3:**
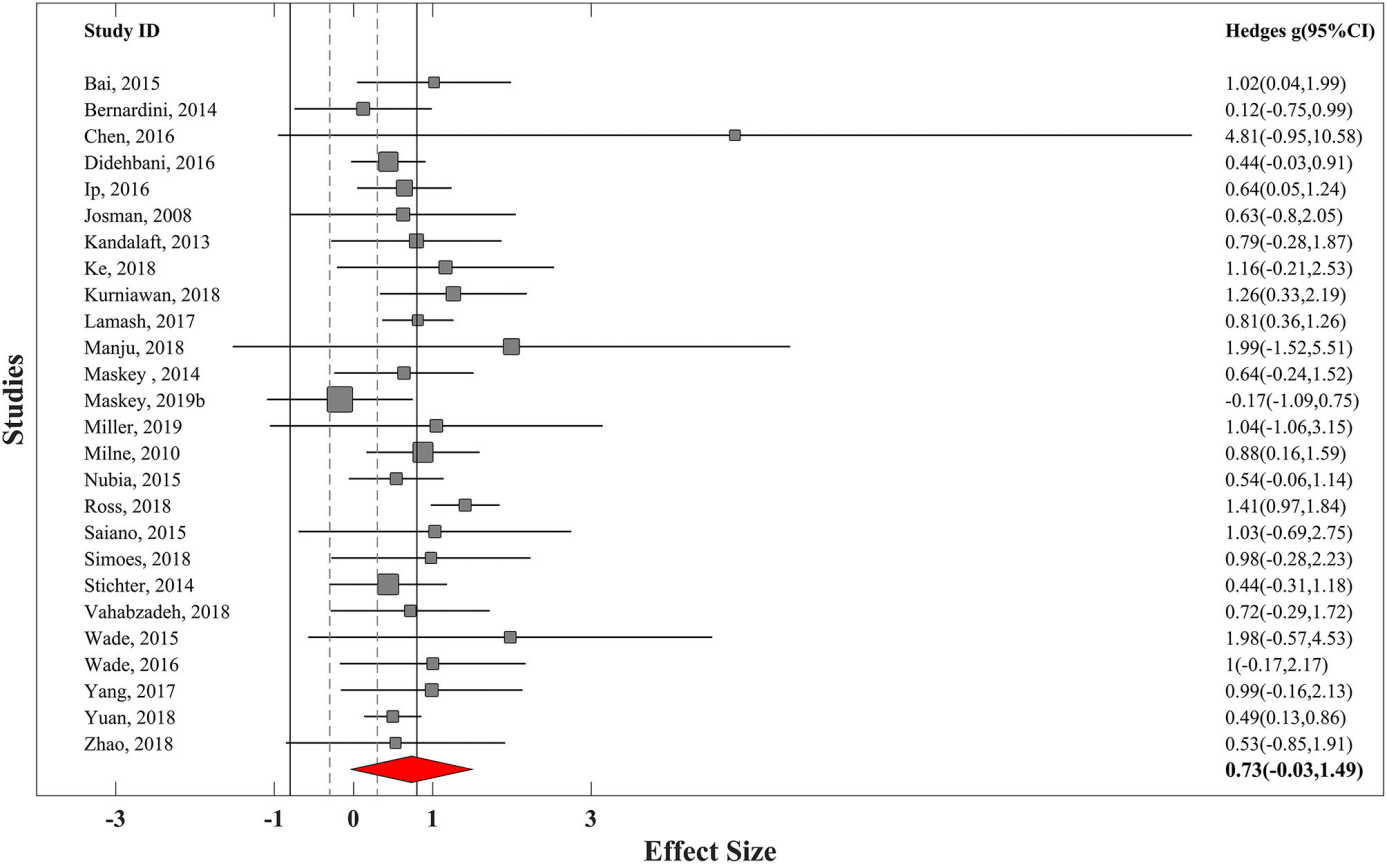
Forest plot of overall effectiveness of VR training for uncontrolled trials with 95% confidence interval. Solid vertical lines represent strong effect size boundary (*g* = −0.8 and 0.8), and dashed vertical lines represent weak effect size boundary (*g* = −0.3 and 0.3).

In the first step, we computed the overall ES for each trial. For nine controlled trials, summary effect size was at medium range (*g* = 0.45, SE_*g*_ = 0.25) with moderate heterogeneity (*I*^2^ = 49.5% and τ^2^ = 0.055). Excluding one of the potential outliers with much larger ES (*g* = 1.8) (97) led to a bit smaller summary ES, but it was still at the medium range of effectiveness (*g* = 0.38, SE_*g*_ = 0.2) (Figure 2). For the 26 uncontrolled trials, a large positive ES was found (*g* = 0.74, SE_*g*_ = 0.17) with low heterogeneity (*I*^2^ = 2.5% and τ^2^ = 0.11). Excluding one of the potential outliers with extremely large ES (*g* = 4.8) (85) led to similar results (*g* = 0.736, SE_*g*_ = 0.17) (Figure 3).

We have interpreted the results for overall effectiveness of studies with random-effects model of meta-analysis relying more on controlled trials because of their more robust experimental design.

### Skill-Based Effectiveness of VR Training

**Figure 4:**
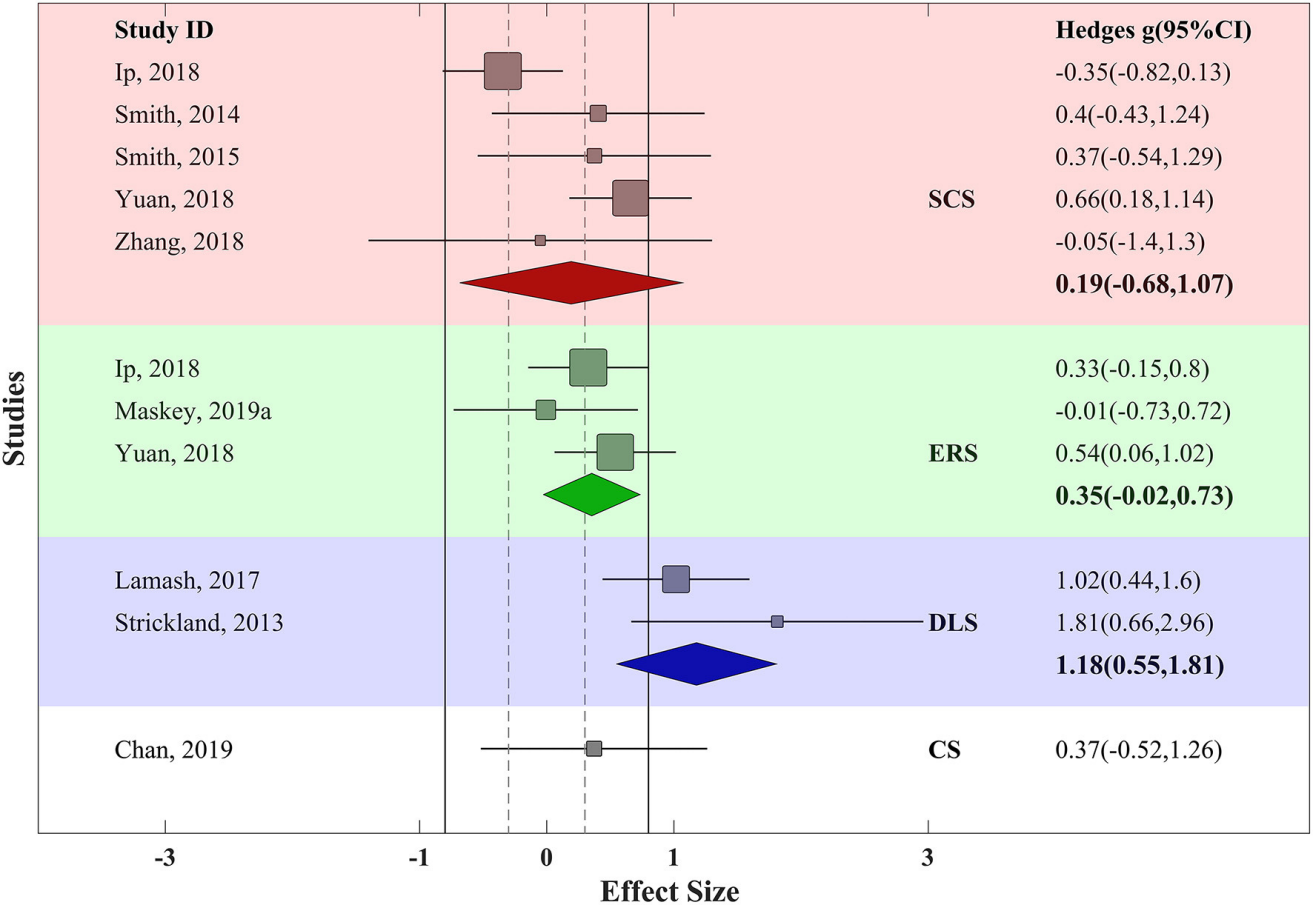
Funnel plot for VR training effectiveness of both uncontrolled and controlled trials with pseudo−95% confidence interval. Red area represents SCS; green area represents ERS; blue area represents DLS; and yellow area represents CS. Solid vertical lines represent strong effect size boundary (*g* = −0.8 and 0.8), and dashed vertical lines represent weak effect size boundary (*g* = −0.3 and 0.3). Filled and empty circles represent Hedges *g* value of uncontrolled and controlled trials, respectively. Solid lines represent *g* with 95% confidence interval of uncontrolled trials and dashed lines represent *g* with 95% confidence interval of controlled trials.

Further, ESs were computed for each skill category (defined in *Methods*). In controlled trials, SCS was addressed in five trials, ERS in three trials, DLS in two trials, and CS in one trial. The effectiveness of VR training was weak for SCS (*g* = 0.2, SE_*g*_ = 0.23, τ^2^ = 0.03), weak to moderate for ERS (*g* = 0.34, SE_*g*_ = 0.06, τ^2^ = 0.02), and again very strong in DLS (*g* = 1.37, SE_*g*_ = 0.18, τ^2^ = 1.12). The single trial that addressed CS revealed weak to moderate effectiveness (*g* = 0.37, SE_*g*_ = 0.002). Regarding heterogeneity in these estimated summary ESs, a considerably large amount of between-study variance (τ^2^) was observed in DLS category in both design groups, but it was relatively small for SCS, ERS, and CS, and it was small for ERS (compare the τ^2^ values presented above) (Figure 4).

**Figure 5:**
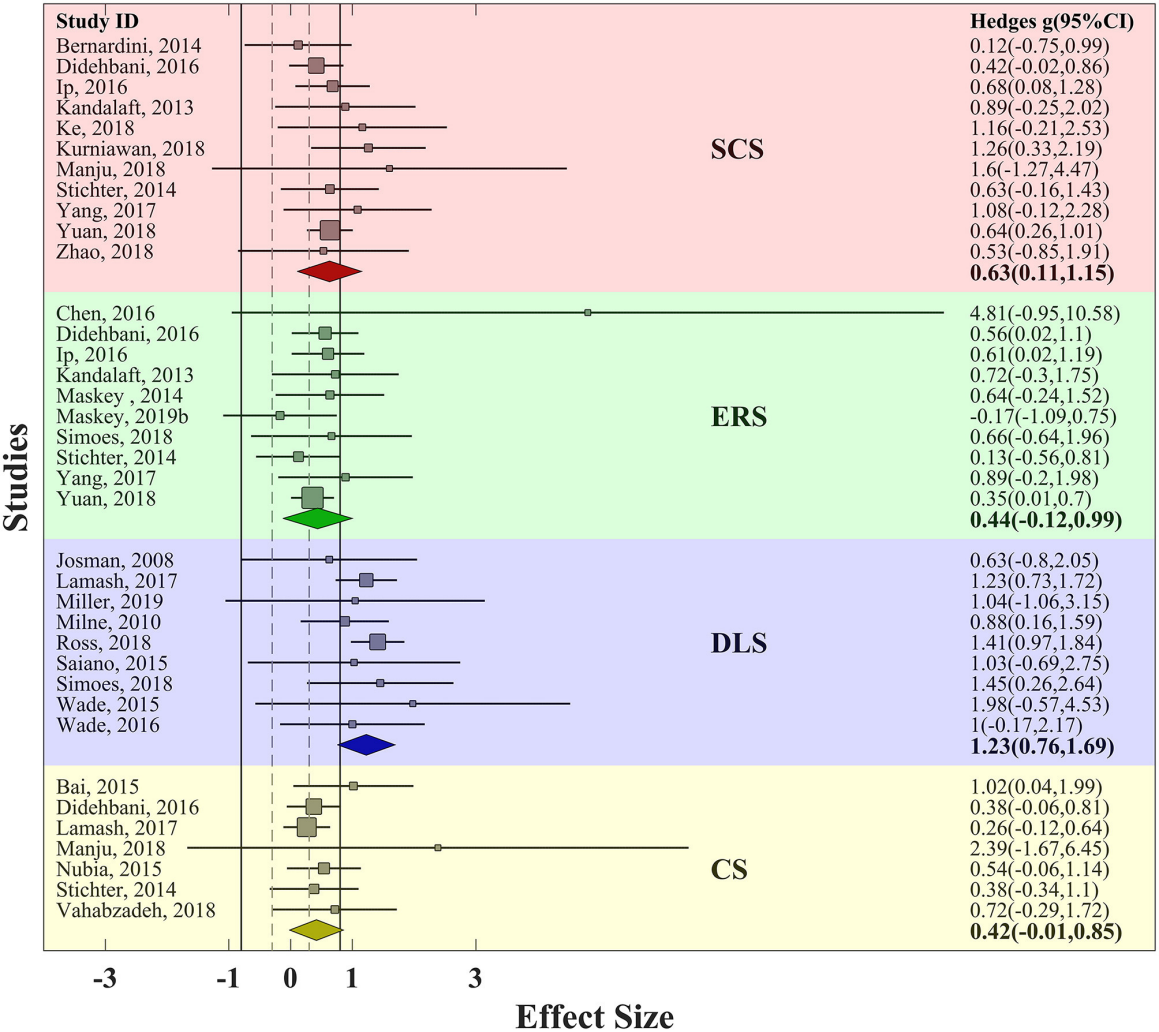
Forest plot of skill-based training effectiveness for uncontrolled trials with 95% confidence interval. Red area represents SCS; green area represents ERS; blue area represents DLS; and yellow area represents CS. Solid vertical lines represent strong effect size boundary (*g* = −0.8 and 0.8), and dashed vertical lines represent weak effect size boundary (*g* = −0.3 and 0.3).

In uncontrolled trials, SCS had been addressed in 11 trials, ERS in 10 trials, DLS in nine trials and CS in seven trials. VR training led to medium to strong effectiveness in SCS (*g* = 0.68, SE_*g*_ = 0.08, τ^2^ = 0.13), medium effectiveness in ERS (*g* = 0.46, SE_*g*_ = 0.05, τ^2^ = 0.07), strong effectiveness in DLS (*g* = 1.16, SE_*g*_ = 0.09, τ^2^ = 0.48), and medium effectiveness in CS (*g* = 0.45, SE_*g*_ = 0.02, τ^2^ = 0.03) (Figure 5). Thus, while in other skills we observe promising effectiveness, the DLS is proven to be the most affected area as its strong effectiveness was consistent among both controlled and uncontrolled trials.

### Analysis of Confounding Factors

As a sizable number of trials had not used any legitimate criteria for screening the participants undergoing intervention (e.g., IQ score, social responsiveness score, disease severity, etc.), we recomputed overall ESs for those trials that screened their population applying this kind of criteria to see how much our results would be biased by this potential confounding factor. In the population screened trials, the results for controlled trials were *g* = 0.25, SE_*g*_ = 0.1, *k* = 7, and τ^2^ = 0.01 and for uncontrolled trials were *g* = 0.72, SE_*g*_ = 0.05, *k* = 12, and τ^2^ = 0.04 and. These results in unscreened trials were *g* = 0.8, SE_*g*_ = 0.05, *k* = 2, and τ^2^ = 0.35 for controlled and *g* = 0.73, SE_*g*_ = 0.2, *k* = 14, and τ^2^ = 0.18 for uncontrolled trials.

Comparing these ESs among two screen groups, the results were not meaningfully different from each other in uncontrolled trials unlike substantial decline from unscreened to screened trials in controlled interventions. Because of very small sample size of unscreened controlled trials, the results derived from them seem less reliable, although more cautions should be devoted to screening population before intervention.

### Subgroup Meta-Analysis

The results of the subgroup analysis for controlled trials would be underpowered and misleading because of its small sample size, so the conclusions of subgroup meta-analysis and meta-regression are limited to the data of uncontrolled trials. By the way, the results of these analyses for controlled trials are available in Supplementary Tables 4, 5.

We performed a subgroup meta-analysis for the categorical moderators described in *Methods*. Results showed that the overall ESs that had been computed based on the data obtained from non-formal measures were somehow larger than those obtained from formal measures (*g* = 0.93 *k* = 11, and τ^2^ = 0.2 for non-formal and *g* = 0.66, *k* = 15, and τ^2^ = 0.09 for formal trials). This can be due to the customized measurements that suit the intervention design, less validity of measures, and susceptibility to the rater bias. Regarding the frequency of each measure's application for categories, most of them had applied formal measures except DLS, which application of non-formal measures was more frequent. In DLS, the summary ES was a bit larger for formal measures, although it was derived from only three trials (*g* = 1.24, *k* = 4, and τ^2^ = 0.55 for formal and *g* = 1.06, *k* = 5, and τ^2^ = 0.28 for non-formal measures). For the type of technology (VR or AR), AR interventions led to a larger overall summary ES (*g* = 0.91, *k* = 5, and τ^2^ = 0.3 for AR and *g* = 0.71, *k* = 21, and τ^2^ = 0.1 for VR). The most of AR interventions were applied for CS that showed more effective training in this skill than VR (*g* = 0.72, *k* = 3, and τ^2^ = 0.15 for AR and *g* = 0.33, *k* = 4, and τ^2^ = 0.01 for VR). Regarding intervention effectiveness for age categories, results showed that skill acquiring, in general, got better as the participants got older (*g* = 0.8, *k* = 4, and τ^2^ = 0.13 for ages 4–8 years; *g* = 0.57, *k* = 7, and τ^2^ = 0.04 for ages 8–12 years; *g* = 0.84, *k* = 7, and τ^2^ = 0.09 for ages 12–16 years; and *g* = 0.85, *k* = 6, and τ^2^ = 0.36 for ages >16 years). Skill categories followed the same trend as the strongest effectiveness observed in the age older than 16 years for all of them (*g* = 0.98, *k* = 2, and τ^2^ = 0.3 for SCS; *g* = 0.46, *k* = 4, and τ^2^ = 0.03 for ERS; and *g* = 1.33, *k* = 3, and τ^2^ = 0.82 for DLS for other age groups; Table 3). It is also noteworthy to say that effectiveness was relatively strong for CS in participants aged 4–8 years, which was the major outcome addressed by our included trials in this age group (*g* = 0.77, *k* = 3, τ^2^ = 0.15). These results point to a more favorable effect of VR interventions for older patients. Subgroup analysis for comorbidity revealed considerable decline in training effectiveness on ASD patients with concomitant comorbidity as *g* = 0.77 in 20 trials with τ^2^ = 0.13 in which patients did not have any specified comorbidity dropped to *g* = 0.6 in six trials with τ^2^ = 0.03 in which patients had some type of comorbidity alongside their main disease. This effect was even more sophisticated in controlled trials so that *g* = 0.57 in six trials with τ^2^ = 0.08 without specified comorbidity reduced to *g* = 0.11 in three trials with τ^2^ = 0.03 in which a comorbidity was diagnosed. The full *Results* can be seen in Table 3.

**Table 3:** Subgroup meta-analysis results for type of measure, type of technology, and age moderators.

**Subgroup**	**Category**	***N***	***g***	**SE*_***g***_***	***Q***	**τ^**2**^**
Formal measure	Overall	15	0.665	0.181	19.7	0.096
	SCS	8	0.604	0.044	3.3	0.107
	ERS	8	0.439	0.052	4.35	0.063
	DLS	4	1.236	0.04	0.701	0.552
	CS	5	0.366	0.007	1.716	0.017
Non-formal measure	Overall	11	0.931	0.097	5.192	0.203
	SCS	3	1.027	0.221	0.773	0.277
	ERS	2	0.957	0.101	1.916	0.241
	DLS	5	1.059	0.156	1.535	0.283
	CS	2	0.719	0.048	0.705	0.141
AR	Overall	5	0.912	0.097	3.975	0.304
	CS	3	0.72	0.039	0.705	0.151
VR	Overall	21	0.715	0.178	21.72	0.099
	SCS	10	0.627	0.047	4.024	0.102
	ERS	9	0.449	0.05	4.481	0.061
	DLS	9	1.155	0.093	3.242	0.477
	CS	4	0.334	0.004	1.173	0.013
Age 4–8 years	Overall	4	0.797	0.059	1.405	0.137
	CS	3	0.775	0.055	1.436	0.149
Age 8–12 years	Overall	7	0.572	0.024	3.457	0.045
	SCS	4	0.582	0.013	0.747	0.12
	ERS	6	0.462	0.028	3.969	0.089
	CS	2	0.377	0.001	2E-04	0.012
Age 12–16 years	Overall	7	0.847	0.06	1.406	0.091
	SCS	2	0.848	0.156	0.402	0.059
	DLS	4	1.11	0.1	1.129	0.295
	CS	2	0.339	0.008	0.704	0.021
Age >16 years	Overall	6	0.854	0.345	11.19	0.356
	SCS	2	0.982	0.041	0.055	0.299
	ERS	4	0.462	0.24	2.752	0.028
	DLS	3	1.331	0.039	0.284	0.825
Comorbidity present	Overall	6	0.608	0.019	1.104	0.033
	ERS	3	0.599	0.002	0.038	0.095
	DLS	3	1.077	0.361	0.771	0.16
	CS	2	0.455	0.011	0.387	0.043
Comorbidity absent or not reported	Overall	20	0.77	0.195	23.67	0.132
	SCS	10	0.744	0.101	4.763	0.143
	ERS	7	0.404	0.06	6.036	0.062
	DLS	6	1.193	0.059	2.208	0.553
	CS	5	0.45	0.03	3.252	0.03

*AR, augmented reality; CS, cognitive skills; DLS, daily living skills; ERS, emotion regulation and recognition skills; g, Hedges g; N, number of trials; Q, Cochrane Q stat; SCS, social and communication skills; SE_g_, standard error of g; VR, virtual reality*.

#### Meta-Regression

To see if there is any significant interaction between continuous moderators (number of sessions, sex, and publication date) and effectiveness of the intervention, we opted for univariate linear regression on weighted ESs as a function of each of these moderators for all designs and skill categories (Table 4). Significant relationship was found in publication date for overall (N = 122, beta1 = 0.4, *p* = 0.02) and DLS (N = 30, beta1 = 0.95, *p* = 0.006), which show that over time, intervention qualities have been likely to be improved as the technology has been advancing; there was no significant association between the number of sessions or gender and computed ESs in any of outcome categories.

**Table 4:** Metaregression results for number of sessions, sex, and publication date moderators.

**Moderator**	**Skill**	***n***	**Slope**	***p***
No. of sessions	Overall	122	−0.035	0.4912
	SCS	38	0.0086	0.8766
	ERS	27	0.0297	0.6552
	DLS	30	0.5425	0.3548
	CS	27	−0.148	0.188
Gender	Overall	122	4.5146	0.2318
	SCS	38	−2.162	0.8148
	ERS	27	6.6054	0.0935
	DLS	30	−0.25	0.9892
	CS	27	−18.7	0.1484
Publication date	Overall^*^	122	0.4021	0.0219^*^
	SCS	38	0.212	0.5024
	ERS	27	−0.237	0.4484
	DLS^*^	30	0.9515	0.0067^*^
	CS	27	0.7987	0.2738

* *Significant values with p < 0.05*.

*CS, cognitive skills; DLS, daily living skills; ERS, emotion regulation and recognition skills; N, number of outcomes in each group; SCS, social and communication skills*.

### Publication Bias

**Figure 6:**
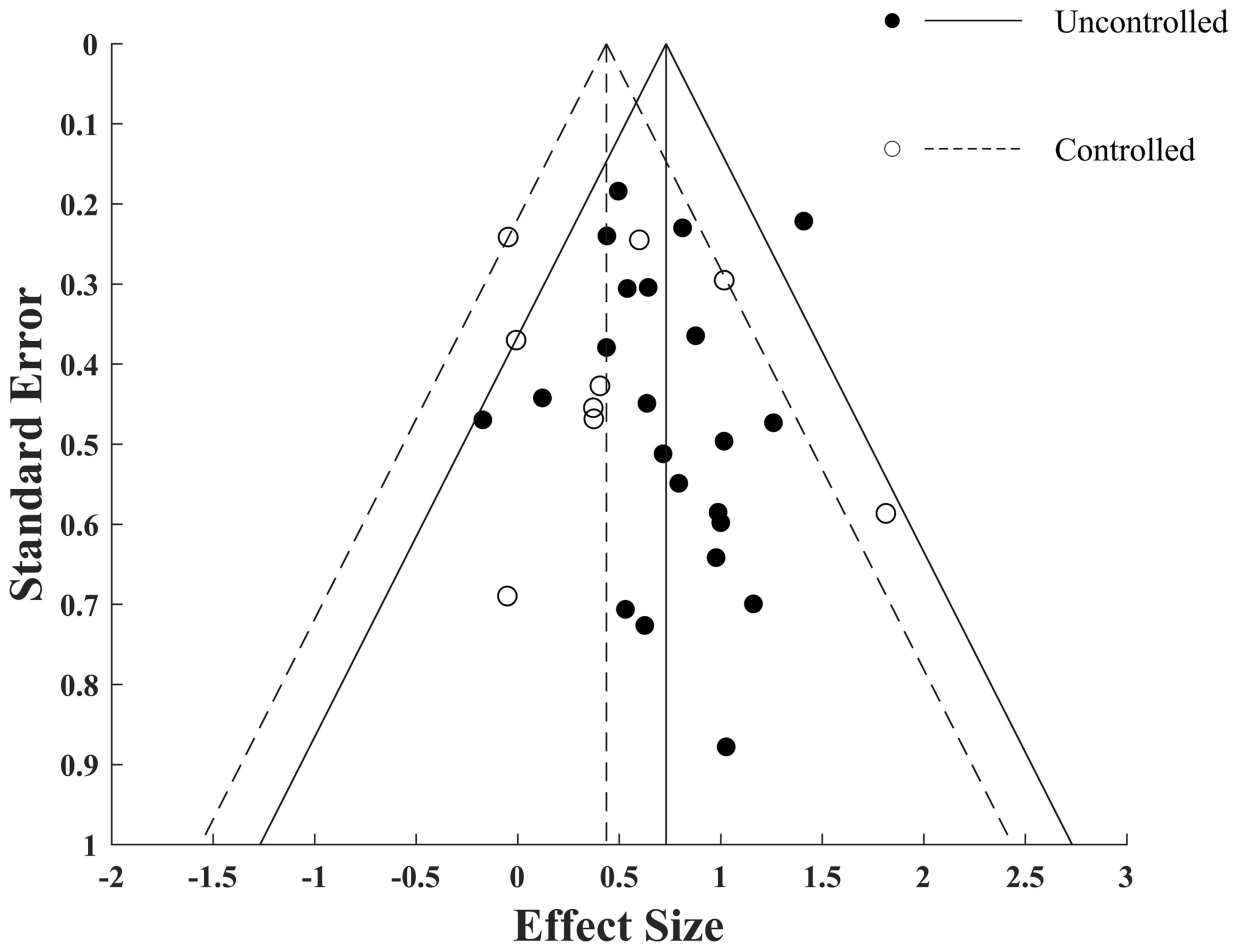
Funnel plot for VR training effectiveness of both uncontrolled and controlled trials with pseudo 95% confidence interval. Filled and empty circles represent Hedges *g* value of uncontrolled and controlled trials respectively. Solid lines represent *g* with 95% confidence interval of uncontrolled trials and dashed lines represent *g* with 95% confidence interval of controlled trials.

Visual inspection of the funnel plot (Figure 6) for both controlled and uncontrolled overall ESs pointed to a symmetrical funnel for both trials. To validate this conclusion statistically, we applied Egger regression intercept test. The test results corroborated the visual inspection by revealing no significant bias for uncontrolled and controlled trials [intercept= 0.27 (*p* = 0.24) for uncontrolled and intercept = 0.1 (*p* = 0.88) for controlled trials]. This implies that drawn conclusions are robust and reliable.

Comparing effectiveness of VR training with some of conventional behavioral programs that were addressed by three meta-analysis studies, we observed a comparable moderate effectiveness of our study with the most of clinical targets appraised by them. One exception was effectiveness of early intensive behavioral intervention on full-scale IQ of patients with ASD, which was proven to be strong (*g* = 1.1 of nine controlled studies). The other interesting finding was that TEACCH and ABA programs were not effective in improving daily living skills (*g* = 0.34 from 6 and *g* = 0.14 from 29 studies, respectively), while the effectiveness of VR training on this clinical target was very strong as it was observed in both controlled and uncontrolled trials. For full results on this part, see Table 5.

**Table 5:** Characteristics and effectiveness of three of the conventional rehabilitation programs.

**References**	**Intervention**	**Publication year**	**No. of studies**	**Case number**	**Study design**	**Outcome measure**	**ES statistic**	**ES**
Virues-Ortega et al. (3)	TEACCH	2013	13	172	Uncontrolled	Overall	Cohen *d*	0.47
			6	93		Eye–hand coordination		0.26
						Motor functioning		0.36
						Gross motor function		0.58
						Imitation		0.41
						Perception		0.4
			5	74		Communication skills		0.34
			6	81		Daily living skills		0.32
			5	74		Social functioning		0.64
			5	43		Cognitive functioning		0.41
			9	121		Verbal skills		0.36
			4	44		Maladaptive behaviors		−0.92
Eldevik et al. (4)	Early Intensive Behavioral	2013	9	153/105 (control)	Controlled	Full-scale IQ	Hedges *g*	1.1
						Adaptive behavior		0.66
Makrygianni et al. (5)	Applied Behavior Analytic interventions	2018	29	831	Uncontrolled	Intellectual abilities	Hedges *g*	0.74
						Communication skills		0.65
						Expressive-language skills		0.742
						Receptive-language skills		0.597
						Non-verbal IQ		0.463
						Adaptive behavior		0.422
						Socialization		0.444
						Daily living skills		0.138

*ES, effect size estimate; TEACCH, Treatment and Education of Autistic and Related Communication Handicapped Children*.

## Discussion

We performed a systematic review and meta-analysis on the effectiveness of applying VR-based therapeutic interventions on the alleviation of deficits in ASD patients. Based on the results of 26 uncontrolled and nine controlled trials, we concluded that VR technology can be a viable tool for designing interventions aimed at enhancing and improving different skills in people suffering from ASD at any age. To our knowledge, this is the first meta-analysis focusing exclusively on the effectiveness of VR-based interventions for training ASD patients. Although there are some meta-analysis studies available on the same topic with various types of new technologies that may (46, 63) or may not (103–105) include VR, the number of trials that methodologically focused on VR was not large enough to draw rigorous conclusions around their efficacy. The increase in the number of VR interventions has been conducted recently; besides, its public availability has justified the need for this study. Overall moderate effectiveness of VR interventions that we observed in this study is in line with the results of previously mentioned studies. Our study shows moderate effectiveness (*g* = 0.44) of VR interventions based on controlled trials and strong effectiveness (*g* = 0.73) based on uncontrolled trials. Although the number of uncontrolled trials was conspicuously larger than controlled ones (26–9), a more credible design of controlled trials leads us to the point to claim moderate effectiveness of VR-based training in individuals with ASD.

Low heterogeneity in uncontrolled trials would provide further support for the conclusion drawn from these trials. Moderate heterogeneity in controlled trials cast doubts on interpretation of their results that could be explained by their relative small sample size (only nine trials comparing to 26 uncontrolled trials) and also the heterogeneity of control groups.

Further analysis of entailed categories of skills revealed relatively the same moderate effectiveness of intervention for SCS, ERS, and CS except daily living skills that outperformed other categories with promising large effectiveness in both design groups. This effect was proven to be consistent across different trials with different designs as heterogeneity was low for both of them. This finding can be specifically of interest because of the more reflective nature of DL skills, which means that they are gained and generalized in later stages of cognitive development, and ASD subjects are required to be trained for it similarly to their neurotypical counterparts. Unlike reflective skills, communication or emotional skills are more intuitive in the sense that they are generally gained in the early stages of development without any specific effort. It is also possible that the observed large effect originated from the larger mean age of participants in this outcome category comparing to other categories, which have given them superiority and dexterity in learning and practicing skills. Nevertheless, significantly larger effectiveness of VR training for DLS compared to others is persuasive enough for us to put forward the hypothesis that reflective skills hold more potential toward improvement by training than intuitive skills. Here we may have corroborated this hypothesis for VR-based training. On the other hand, medium effectiveness for communication, emotion, and CS may be due to the complex mental nature of these skills. As it is reflected in the recent systematic review (62), although these skills have been the center of attention in most studies, just partial improvements have been made. Knowing this, in future efforts, a more elaborated intervention design seems necessary to ascertain the effectiveness of VR training on these types of skills. Taken together, we encourage psychiatrists and educators of people with ASD to practice this type of technology with more focus on daily living skills.

The results of our subgroup analysis are merely discussed on overall outcomes of uncontrolled trials. The small sample size of other subgroups precluded us from drawing a strong conclusion for them. The effectiveness of an intervention based on formal and non-formal measures was roughly similar and around moderate range, which indicates that our results might not be affected with the existence of non-formal assessments. Apart from that, cautious interpretation of informal measures should be considered, and it is possible that defined informal measures could be biased. The effectiveness of AR was similar and even a little larger than VR interventions. Although the sample size of the AR subgroup was relatively small, considering its low heterogeneity, the resultant conclusion on this subgroup can be reliable. This is particularly important because AR interventions can be conducted by means of AR-enabled mobile phones, which is ubiquitous nowadays providing more controlled interventions for larger populations of patients with ASD. The superiority of AR can be assigned to its simplicity in design and convenience of use compared to VR in which tasks are designed and applied in more complex environments with more parameters to understand and deal with. This simplicity can lead to a sooner and better engagement of participants in the task specifically for younger children.

The results of the subgroup analysis for age categories revealed that performance improves as the age gets larger. Particularly, it is important to note that this improvement is happening not only in daily living skills, which are reflective skills and later in development, but also in other intuitive areas, such as social skills and emotion recognition skills. This phenomenon may be induced by two factors. First, patients with autism presumably develop a kind of mechanism to overcome the deficits primarily caused by ASD, and so they assimilate to their milieu as they age. Second, older patients may have the advantage that they understand the task and VR environment better, and so they interact with it more efficiently, resulting in improved performance. In the first age category (4–8 years old), a notable relatively strong effectiveness was observed in CS, which was the only addressed area in this age group. Despite the small sample size, the relatively large ES was persuasive enough for us to consider it. This large effect may also be seen in other areas of SCS, ERS, and DLS; therefore, we encourage the scientific community to target their interventions on these areas too.

We observed a substantial decline in the effectiveness of training on patients who suffered from some sort of concomitant comorbidity along with ASD. This phenomenon was particularly interesting in controlled trials as observed moderate effectiveness of training on ASD patients without other comorbidity was completely vanished when concomitant comorbidity was taken into account. This alarms the future practitioners who are trying to improve skills in patients with ASD by means of VR interventions to carefully screen their target patients for having other concomitant comorbidity.

The effectiveness of training for HFASD patients was moderate to large, which was equivalent to its overall value regardless of the level of the disorder. In most of the studies, whether composed of a combination of LFASD and HFASD participants or the level of disease was not specified, direct association between level of disease and effectiveness of intervention could not be derived. For this reason, we call for papers with more focus on defining the level and functionality of disease in included participants for better characterizing the target population of intervention.

The results of meta-regression revealed a significant correlation between publication date and VR training effectiveness, which can be interpreted under improvement in the design and conduction of VR interventions over time. Surprisingly, effectiveness was not influenced by the total number of intervention sessions. It is important to mind that the session's duration and its distribution over the course of intervention were unreported or highly heterogeneous among the trials, and therefore, the net number of sessions might not be a good representative for intensity and quality of intervention. For this reason, hesitant interpretations warrant caution, and more controlled interventions in terms of design, duration, and longevity are needed for more conclusive interpretations on this matter. The sex of participants was not a significant moderator of the results in our study as it is not seen in other studies of this kind.

Comparing the results of the current meta-analysis with those of more conventional training programs (Table 5), it is evident that VR-based training is at least as effective in most study endpoints as traditional programs. In addition, the more flexible and favorable nature of VR leads to more elaborate task designs, more enthusiasm in participants to do those tasks, and ultimately more accurate assessments of improvement. These factors together might result in more ecological validity of VR-based experiments and more reliability of their results.

The strong effectiveness of daily living skills (reflected in both controlled and uncontrolled trials) was achieved only through VR-based training, not conventional training. It is therefore sensible to use VR to design rehabilitation programs aimed at daily living skills in clinical practice. In the other clinical targets, a further improvement in the design and application of VR technology is still required.

### Limitations and Recommendations for Future Research

Most of our included studies were uncontrolled pretest–posttest trials. It has been argued that these types of trials should be avoided in meta-analysis as the pretest and posttest scores are not independent of each other, and thus, accurate calculation of SMDs requires knowledge of correlation value between these two scores, which is not provided in most of the studies (106). Aside from that, perhaps due to differing epistemological bases of research being carried out in this broad domain, most of the studies have adopted this type of design for their intervention, which makes considering this massive body of data for analysis inevitable. Here, we have done all the calculations with the premise of independent pretest and posttest scores (zero correlation), which is subsequently leading to the largest pooled variance and thus the smallest possible value of computed ES. For this reason, we claim that our applied method is the most parsimonious one avoiding any overestimation in computing the ESs.

Although the number of participants in most studies was rather low, and so their estimations would not be adequately powered, its effect might be compensated by a considerably large number of included trials. Many trials had not screened participants for critical contributing factors that could affect the outcome. This issue seemed to be a challenge for our results. However, later analysis relieved this by showing that the summary ES of those trials that screened included population did not deviate drastically from those who did not perform this screening.

The type of VR technology applied by studies was diverse enough to prevent us from establishing a systematic relationship between the technology type and its effectiveness, so further studies are required to investigate such a connection. To our surprise, restricted and repetitive behavior, which is one of the core symptoms of ASD, was not addressed by any of studies, so more experiments are encouraged to be targeted in this area in the future works. Follow-up assessment of participants was performed in an only limited number of trials; therefore, the maintenance of treatment effects, although important, could not be assessed in our study.

## Conclusions

The current findings support the effectiveness of VR training to improve ASD-related disabilities. The strong observed effectiveness for daily living skills could justify the application of VR interventions in clinical practice. For future research, the designed experiments need to be more controlled in terms of selection of participants, type and duration of intervention, and choice of a measurement tool, and finally, more efforts should be devoted to follow-up assessments carried out weeks or months after the end of the intervention to ensure that the effects of training are consolidated and maintained.

## Data Availability Statement

The original contributions presented in the study are included in the article/Supplementary Material, further inquiries can be directed to the corresponding author/s.

## Author Contributions

A-HV and FA designed the study and wrote the protocol. BK, RK, and MR conducted literature searches and provided summaries of previous research studies. BK analyzed data and produced figures. BK and RK wrote the first draft of the manuscript. A-HV supervised the study. All authors contributed to and have approved the final manuscript.

## Conflict of Interest

The authors declare that the research was conducted in the absence of any commercial or financial relationships that could be construed as a potential conflict of interest.
