# Supplementary material for: Effectiveness of Virtual/Augmented Reality–Based Therapeutic Interventions on Individuals With Autism Spectrum Disorder: A Comprehensive Meta-Analysis
Source: Front Psychiatry. 2021 Jun 23;12:665326. doi: 10.3389/fpsyt.2021.665326 (PMC8260941; doi:10.3389/fpsyt.2021.665326)
Supplement: Supplementary file 1 [file Data_Sheet_1.PDF]

1. Search strategy with adjusted queries for each database

Pubmed:

1) "virtual reality" OR "virtual realit*" OR "virtual-reality" OR "virtual-realit*" OR "VR" OR "VRET "OR "VRT "OR "virtual environment" OR "virtual environment*" OR "VE" OR "virtual world" OR "virtual world*" OR "virtual-world "OR "virtual-world* "OR "computer world" OR "computer-world" OR "computer world*" OR "computer-world*" OR "computer simulated environment" OR "computer simulated environments*" OR "computer-simulated-environment" OR "computer-simulated-environment*" OR "comput* simulat* environment*" OR "artificial reality" OR "artificial realit*" OR "artificial-reality" OR "artificial-realit*" OR "mixed reality" OR "mix* reality" OR "mix* realit*" OR "mediated reality" OR "mediated realit*" OR "mediat* realit*" OR "augmented reality" OR "augmented realit*" OR "augmented-reality" OR "augmented-realit*" OR "augment* realit*"
2) Wii OR Kinect OR Nintendo OR "augment* gam* "OR "Virtual gam* "OR "virtual-game "OR "virtual-gam*"
3) "smartglasses" OR "smartglass*" OR "smart glasses" OR "smart glass*" OR "smart-glasses" OR "smart-glass*" OR "3D glasses" OR "3D glass*"
4) "Virtual Reality"[Mesh] OR "Virtual Reality Exposure Therapy"[Mesh] OR "Virtual Reality Exposure Therapy/education"[Mesh] OR "Simulation Training"[Mesh] OR “Video games” [mesh] OR "Video Games/therapeutic use"[Mesh]
5) 1 OR 2 OR 3 OR 4
6) "Autism "OR "autistic "OR "autis* "OR "Asperger "OR "asperger’s "OR "Asperger* "OR "autism spectrum disorder "OR "high function autism "OR "high function ASD "OR "Asperger syndrome "OR "pervasive developmental "OR "pervasive development" OR "pervasive development*" OR "PDDNOS"
7) "Autistic Disorder"[Mesh] OR "Autism Spectrum Disorder"[Mesh] AND "Asperger Syndrome"[Mesh] OR "Child Development Disorders, Pervasive"[Mesh]
8) 6 OR 7
9) 5 AND 8

ERIC:

1) "virtual reality" OR "virtual-reality" OR "VR" OR "VRET "OR "VRT "OR "virtual environment" OR "virtual environments" OR "VE" OR "virtual world" OR "virtual worlds" OR "virtual-world " OR "virtual-worlds "OR "computer world" OR "computer-world" OR "computer worlds" OR "computer-worlds" OR "computer simulated environment" OR "computer simulated environments" OR "computer-simulated-environment" OR "computer-simulated-environments" OR "artificial reality" OR "artificial realities" OR "artificial-reality" OR "artificial-realities" OR "mixed reality" OR "mixed realities" OR "mediated reality" OR "mediated realities" OR "augmented reality" OR "augmented realities" OR "augmented-reality" OR "augmented-realities"
2) Wii OR Kinect OR Nintendo OR "augmented game" OR "augmented games" OR "augmented gaming" OR "Virtual game" OR "virtual games" OR "virtual gaming"
3) "smartglasses" OR "smart glasses" OR "smart-glasses" OR "3D glasses" OR "3Dglasses" OR "3D-glasses"
4) 1 OR 2 OR 3

5) "Autism "OR "autistic "OR "Asperger "OR "asperger’s "OR "autism spectrum disorder "OR "high function autism "OR "high function ASD "OR "Asperger syndrome "OR "pervasive developmental "OR "pervasive development" OR "PDDNOS"
6) 4 AND 5

PsycINFO

1) "virtual reality" OR "virtual realit*" OR "virtual-reality" OR "virtual-realit*" OR "VR" OR "VRET "OR "VRT "OR "virtual environment" OR "virtual environment*" OR "VE" OR "virtual world" OR "virtual world*" OR "virtual-world "OR "virtual-world* "OR "computer world" OR "computer-world" OR "computer world*" OR "computer-world*" OR "computer simulated environment" OR "computer simulated environment*" OR "computer-simulated-environment" OR "computer-simulated-environment*" OR "comput* simulat* environment*" OR "artificial reality" OR "artificial realit*" OR "artificial-reality" OR "artificial-realit*" OR "mixed reality" OR "mix* reality" OR "mix* realit*" OR "mediated reality" OR "mediated realit*" OR "mediat* realit*" OR "augmented reality" OR "augmented realit*" OR "augmented-reality" OR "augmented-realit*" OR "augment* realit*"
2) Wii OR Kinect OR Nintendo OR "augment* gam* "OR "Virtual gam* "OR "virtual-game "OR "virtual-gam*"
3) "smartglasses" OR "smart glasses" OR "smart-glasses" OR "3D glasses" OR "3Dglasses" OR "3D-glasses"
4) 1 OR 2 OR 3
5) "Autism "OR "autistic "OR "autis* "OR "Asperger "OR "asperger’s "OR "Asperger* "OR "autism spectrum disorder "OR "high function autism "OR "high function ASD "OR "Asperger syndrome "OR "pervasive developmental "OR "pervasive development" OR "pervasive development*" OR "PDDNOS"
6) 4 AND 5

IEEE

1) "virtual reality" OR "virtual-reality" OR "VR" OR "VRET" OR "VRT" OR "virtual environment" OR "VE" OR "virtual world" OR "virtual-world" OR "computer world" OR "computer-world" OR "computer simulated environment" OR "computer-simulated-environment" OR "artificial reality" OR "artificial-reality" OR "mixed reality" OR "mixed realities" OR "mediated reality" OR "augmented reality" OR "augmented-reality"
2) Wii OR Kinect OR Nintendo OR "augmented gam*" OR "Virtual gam*" OR "virtual-game" OR "virtual-gam*"
3) "smartglasses" OR "smart glasses" OR "smart-glasses" OR "3D glasses" OR "3Dglasses" OR "3D-glasses"
4) 1 OR 2 OR 3
5) "Autism" OR "autistic" OR "Asperger" OR "asperger’s" OR "autism spectrum disorder" OR "high function autism" OR "high function ASD" OR "Asperger syndrome" OR "pervasive developmental" OR "pervasive development" OR "PDDNOS"
6) 4 AND 5

Web of Science:

1) "virtual reality" OR "virtual realit*" OR "virtual-reality" OR "virtual-realit*" OR "VR" OR "VRET "OR "VRT "OR "virtual environment" OR "virtual environment*" OR "VE" OR "virtual world" OR "virtual world*" OR "virtual-world "OR "virtual-world* "OR "computer world" OR "computer-world" OR "computer world*" OR "computer-world*" OR "computer simulated environment" OR "computer simulated environments*" OR "computer-simulated-environment" OR "computer-simulated-environment*" OR "comput* simulat* environment*" OR "artificial reality" OR "artificial realit*" OR "artificial-reality" OR "artificial-realit*" OR "mixed reality" OR "mix* reality" OR "mix* realit*" OR "mediated reality" OR "mediated realit*" OR "mediat* realit*" OR "augmented reality" OR "augmented realit*" OR "augmented-reality" OR "augmented-realit*" OR "augment* realit*"

2) Wii OR Kinect OR Nintendo OR "augment* gam* "OR "Virtual gam* "OR "virtual-game "OR "virtual-gam*"
3) "smartglasses" OR "smart glasses" OR "smart-glasses" OR "3D glasses" OR "3Dglasses" OR "3D-glasses"
4) (virtual NEAR/2 realit*) OR (virtual NEAR/2 environment*) OR (virtual NEAR/2 world*) OR (artifial NEAR/2 realit*) OR (mix* NEAR/2 realit*) OR (mediated NEAR/2 realit*) OR (augment* NEAR/2 realit*) OR (augment* NEAR/2 gam*) OR (virtual NEAR/2 gam*)
5) 1 OR 2 OR 3 OR 4
6) "Autism "OR "autistic "OR "autis* "OR "Asperger "OR "asperger’s "OR "Asperger* "OR "autism spectrum disorder "OR "high function autism "OR "high function ASD "OR "Asperger syndrome "OR "pervasive developmental disorder "OR "pervasive development disorder" OR "pervasive development* disorder" OR "PDDNOS"
7) (pervasive NEAR/2 development* NEAR/2 disorder)
8) 6 OR 7
9) 5 AND 5

2. Sensitivity analysis on the choice of rho

Table 1: **Sensitivity analysis on the choice of correlation value (rho) in Robust Variance Estimation method.**

outcome category	intervention design		b_{1j}	V_R	tau2	b_{1j}	V_R	tau2	b_{1j}	V_R	tau2	b_{1j}	V_R	tau2	b_{1j}	V_R	tau2
		k	rho=0			rho=0.1			rho=0.2			rho=0.3			rho=0.4		
Overall	uncontrolled	26	0.74	0.17	0.11	0.79	0.2	0.14	0.81	0.21	0.16	0.83	0.22	0.16	0.84	0.23	0.17
	controlled	9	0.45	0.25	0.06	0.55	0.36	0.08	0.6	0.42	0.1	0.63	0.46	0.11	0.65	0.49	0.12
SCS	uncontrolled	11	0.69	0.08	0.13	0.71	0.09	0.15	0.73	0.1	0.16	0.74	0.11	0.17	0.76	0.11	0.18
	controlled	5	0.2	0.23	0.03	0.25	0.27	0.04	0.29	0.28	0.04	0.33	0.3	0.04	0.35	0.31	0.05
ERS	uncontrolled	10	0.46	0.05	0.07	0.48	0.05	0.07	0.49	0.04	0.07	0.5	0.04	0.07	0.5	0.04	0.07
	controlled	3	0.34	0.06	0.02	0.38	0.08	0.03	0.4	0.09	0.04	0.42	0.09	0.04	0.43	0.1	0.04
DLS	uncontrolled	9	1.16	0.09	0.48	1.18	0.1	0.5	1.19	0.11	0.52	1.2	0.11	0.52	1.21	0.12	0.52
	controlled	2	1.38	0.18	1.12	1.36	0.16	1.11	1.34	0.15	1.09	1.32	0.13	1.08	1.31	0.12	1.07
CS	uncontrolled	7	0.45	0.02	0.03	0.46	0.03	0.03	0.46	0.03	0.03	0.47	0.03	0.03	0.47	0.03	0.03
			rho=0.5			rho=0.6			rho=0.7			rho=0.8			rho=1		
Overall	uncontrolled	26	0.85	0.24	0.17	0.86	0.24	0.17	0.86	0.25	0.17	0.87	0.25	0.17	0.88	0.26	0.16
	controlled	9	0.67	0.51	0.13	0.69	0.53	0.14	0.71	0.54	0.14	0.72	0.56	0.15	0.74	0.58	0.16
SCS	uncontrolled	11	0.77	0.12	0.19	0.78	0.12	0.19	0.78	0.13	0.2	0.79	0.13	0.2	0.8	0.14	0.21
	controlled	5	0.38	0.31	0.05	0.4	0.32	0.05	0.41	0.32	0.06	0.43	0.32	0.06	0.45	0.33	0.06
ERS	uncontrolled	10	0.51	0.03	0.07	0.51	0.03	0.07	0.51	0.03	0.07	0.51	0.03	0.07	0.51	0.03	0.06
	controlled	3	0.44	0.1	0.05	0.45	0.1	0.05	0.46	0.11	0.05	0.46	0.11	0.05	0.47	0.11	0.06
DLS	uncontrolled	9	1.22	0.12	0.53	1.23	0.13	0.52	1.23	0.14	0.52	1.24	0.14	0.52	1.25	0.15	0.51
	controlled	2	1.3	0.11	1.06	1.29	0.1	1.05	1.27	0.1	1.04	1.26	0.09	1.03	1.25	0.08	1.02
CS	uncontrolled	7	0.47	0.03	0.02	0.47	0.03	0.02	0.47	0.03	0.02	0.47	0.03	0.01	0.47	0.03	0.01

k: number of trials, rho: within-study correlation, b1j: estimated summary effect size of studies, VR: estimated robust variance, tau2: estimated between study variance

3. Three-level meta-analysis

First the data were structured in a table and estimating summary effect size, between and within-study variance and heterogeneity measures was considered by using meta3 function of metaSEM R package. In what follows, we have provided a sample of tabular structure of input data, R scripts of three-level meta-analysis with and without considering skill category as a moderator, and the table of results of the analysis.

Table 2: an example for tabular structure of input data for meta3 function

Study	SCS	ERS	DLS	CS	g	v
Manju, 2018	0	0	0	1	2.39	4.29
Manju, 2018	1	0	0	0	1.6	2.15
Kurniawan, 2018	1	0	0	0	1.26	0.22
Chen, 2016	0	1	0	0	4.81	8.66
Didehbani, 2016	0	1	0	0	0.66	0.06
Didehbani, 2016	0	1	0	0	0.46	0.1
Didehbani, 2016	1	0	0	0	0.38	0.05
Didehbani, 2016	1	0	0	0	0.45	0.05
Didehbani, 2016	0	0	0	1	0.18	0.04
Didehbani, 2016	0	0	0	1	0.42	0.04
Didehbani, 2016	0	0	0	1	0.52	0.07
Ip, 2016	0	1	0	0	0.53	0.09
Ip, 2016	0	1	0	0	0.68	0.09
Ip, 2016	1	0	0	0	0.6	0.09
Ip, 2016	1	0	0	0	0.76	0.1
Maskey , 2014	0	1	0	0	0.62	0.2
Maskey , 2014	0	1	0	0	0.66	0.2
…						

SCS: social and communication skills, ERS: emotion recognition and regulation skills, DLS: daily living skills, CS: cognitive skills, g: Hedges g effect size of outcome, v: effect size sampling error.

Analysis without moderator, Overall effectiveness of uncontrolled trials

```
Call:
metaSEM::meta3(y = g, v = v, cluster = Study, data = Uncontrolled_Overall_Cat
)

95% confidence intervals: z statistic approximation (robust=FALSE)
Coefficients:
            Estimate  Std.Error     lbound     ubound z value  Pr(>|z|)
Intercept  0.6327728  0.0781771  0.4795485  0.7859970  8.0941 6.661e-16 ***
Tau2_2     0.0535696  0.0219523  0.0105437  0.0965954  2.4403   0.01468 *
Tau2_3     0.0624055  0.0352684 -0.0067193  0.1315303  1.7694   0.07682 .
---
Signif. codes:  0 ‘***’ 0.001 ‘**’ 0.01 ‘*’ 0.05 ‘.’ 0.1 ‘ ’ 1

Q statistic on the homogeneity of effect sizes: 195.924
Degrees of freedom of the Q statistic: 121
P value of the Q statistic: 1.923339e-05

Heterogeneity indices (based on the estimated Tau2):
```

```
                              Estimate
I2_2 (Typical v: Q statistic)   0.2096
I2_3 (Typical v: Q statistic)   0.2442

Number of studies (or clusters): 26
Number of observed statistics: 122
Number of estimated parameters: 3
Degrees of freedom: 119
-2 log likelihood: 189.2395
OpenMx status1: 0 ("0" or "1": The optimization is considered fine.
Other values may indicate problems.)
```

Analysis without moderator, Overall effectiveness of uncontrolled trials

```
Call:
metaSEM::meta3(y = g, v = v, cluster = Study, data = Controlled_Overall_Cat)

95% confidence intervals: z statistic approximation (robust=FALSE)
Coefficients:
           Estimate Std.Error    lbound    ubound z value Pr(>|z|)
Intercept  0.374326  0.143153  0.093751  0.654901  2.6149 0.008926 **
Tau2_2     0.029928  0.028621 -0.026168  0.086023  1.0457 0.295715
Tau2_3     0.106316  0.087206 -0.064604  0.277235  1.2191 0.222792
---
Signif. codes:  0 ‘***’ 0.001 ‘**’ 0.01 ‘*’ 0.05 ‘.’ 0.1 ‘ ’ 1

Q statistic on the homogeneity of effect sizes: 80.06554
Degrees of freedom of the Q statistic: 44
P value of the Q statistic: 0.0007221185

Heterogeneity indices (based on the estimated Tau2):
                              Estimate
I2_2 (Typical v: Q statistic)   0.1163
I2_3 (Typical v: Q statistic)   0.4131

Number of studies (or clusters): 9
Number of observed statistics: 45
Number of estimated parameters: 3
Degrees of freedom: 42
-2 log likelihood: 57.26877
OpenMx status1: 0 ("0" or "1": The optimization is considered fine.
Other values may indicate problems.)
```

Analysis with skill categories as moderators, uncontrolled trials

```
Call:
metaSEM::meta3(y = g, v = v, cluster = Study, x = cbind(SCS,
    ERS, DLS, CS), data = Uncontrolled_Overall_Cat, intercept.constraints = 0
)

95% confidence intervals: z statistic approximation (robust=FALSE)
Coefficients:
          Estimate  Std.Error     lbound     ubound z value  Pr(>|z|)
Slope_1  0.5687535  0.0963226  0.3799647  0.7575423  5.9047 3.533e-09 ***
Slope_2  0.3979478  0.1041272  0.1938622  0.6020334  3.8217 0.0001325 ***
Slope_3  1.0371415  0.1144312  0.8128605  1.2614224  9.0635 < 2.2e-16 ***
Slope_4  0.4176660  0.0966452  0.2282449  0.6070871  4.3216 1.549e-05 ***
Tau2_2   0.0240959  0.0163095 -0.0078702  0.0560620  1.4774 0.1395646
Tau2_3   0.0367565  0.0286448 -0.0193862  0.0928992  1.2832 0.1994280
---
Signif. codes:  0 ‘***’ 0.001 ‘**’ 0.01 ‘*’ 0.05 ‘.’ 0.1 ‘ ’ 1
```

```
Q statistic on the homogeneity of effect sizes: 195.924
Degrees of freedom of the Q statistic: 121
P value of the Q statistic: 1.923339e-05

Explained variances (R2):
                        Level 2 Level 3
Tau2 (no predictor)    0.053570  0.0624
Tau2 (with predictors) 0.024096  0.0368
R2                     0.550194  0.4110

Number of studies (or clusters): 26
Number of observed statistics: 122
Number of estimated parameters: 6
Degrees of freedom: 116
-2 log likelihood: 167.722
OpenMx status1: 0 ("0" or "1": The optimization is considered fine.
Other values may indicate problems.)
```

Analysis with skill categories as moderators, controlled trials

```
Call:
metaSEM::meta3(y = g, v = v, cluster = Study, x = cbind(SCS,
    ERS, DLS, CS), data = Controlled_Overall_Cat, intercept.constraints = 0)

95% confidence intervals: z statistic approximation (robust=FALSE)
Coefficients:
           Estimate   Std.Error      lbound      ubound z value  Pr(>|z|)
Slope_1  4.9307e-02  1.4549e-01 -2.3585e-01  3.3447e-01  0.3389 0.7346863
Slope_2  4.6463e-01  1.7096e-01  1.2956e-01  7.9970e-01  2.7178 0.0065719 **
Slope_3  1.2044e+00  3.1024e-01  5.9630e-01  1.8124e+00  3.8821 0.0001036 ***
Slope_4  3.7208e-01  4.2506e-01 -4.6102e-01  1.2052e+00  0.8754 0.3813773
Tau2_2   1.0000e-10  2.9532e-02 -5.7881e-02  5.7881e-02  0.0000 1.0000000
Tau2_3   7.7378e-02  5.3677e-02 -2.7827e-02  1.8258e-01  1.4416 0.1494287
---
Signif. codes:  0 ‘***’ 0.001 ‘**’ 0.01 ‘*’ 0.05 ‘.’ 0.1 ‘ ’ 1

Q statistic on the homogeneity of effect sizes: 80.06554
Degrees of freedom of the Q statistic: 44
P value of the Q statistic: 0.0007221185

Explained variances (R2):
                          Level 2 Level 3
Tau2 (no predictor)    2.9928e-02  0.1063
Tau2 (with predictors) 1.0000e-10  0.0774
R2                     1.0000e+00  0.2722

Number of studies (or clusters): 9
Number of observed statistics: 45
Number of estimated parameters: 6
Degrees of freedom: 39
-2 log likelihood: 41.29098
OpenMx status1: 0 ("0" or "1": The optimization is considered fine.
Other values may indicate problems.)
```

Table 3: three-level meta-analysis results

<table>
<tr><th>Design</th><th>category</th><th>g_m</th><th>SE</th><th>tau2_2</th><th>tau2_3</th><th>Q</th></tr>
<tr><td rowspan="5">Uncontrolled</td><td>Overall</td><td>0.63</td><td>0.08</td><td>0.0536</td><td>0.0624</td><td>195.92</td></tr>
<tr><td>SCS</td><td>0.57</td><td>0.1</td><td rowspan="4">0.0241</td><td rowspan="4">0.0368</td><td rowspan="4">195.92</td></tr>
<tr><td>ERS</td><td>0.4</td><td>0.1</td></tr>
<tr><td>DLS</td><td>1.04</td><td>0.11</td></tr>
<tr><td>CS</td><td>0.42</td><td>0.1</td></tr>
<tr><td rowspan="4">Controlled</td><td>Overall</td><td>0.37</td><td>0.14</td><td>0.0299</td><td>0.1063</td><td>80.066</td></tr>
<tr><td>SCS</td><td>0.05</td><td>0.15</td><td rowspan="3">0</td><td rowspan="3">0.0774</td><td rowspan="3">80.066</td></tr>
<tr><td>ERS</td><td>0.46</td><td>0.17</td></tr>
<tr><td>DLS</td><td>1.2</td><td>0.31</td></tr>
</table>

SCS: social and communication skills, ERS: emotion recognition and regulation skills, DLS: daily living skills, CS: cognitive skills, gm: average effect size estimate, SE: standard error of gm, tau2_2: between-study variance, tau2_3: within-study variance. Q: statistic on the homogeneity of effect sizes

4. Controlled trials subgroup meta-analysis and meta-regression

Table 4: subgroup meta-analysis results of controlled trials

subgroup	category	N	g	SEg	Q	I2	tau2
formal	Overall	7	0.38	0.22	10.07	40.42	0.035
	SCS	5	0.2	0.23	9.094	56.01	0.03
	ERS	3	0.34	0.06	1.548	0	0.025
non-formal	Overall	2	1.03	0.53	3.883	74.25	0.531
age: 4-8	Overall	2	0.27	0.04	0.266	0	0.073
age: 8-12	Overall	2	0.38	0.14	1.898	47.32	0.04
	ERS	2	0.35	0.16	1.539	35.04	0.035
age: 12-16	Overall	2	0.43	0.39	7.844	87.25	0.06
age: >16	Overall	3	0.75	0.33	4.581	56.34	0.089
	SCS	2	0.39	0	0.002	0	0.005
Comorbidity present	Overall	3	0.11	0.03	0.488	0	0.028
Comorbidity absent or not reported	Overall	6	0.57	0.28	13.67	63.42	0.079
	SCS	4	0.22	0.23	8.971	66.56	0.036
	ERS	2	0.43	0.02	0.383	0	0.055
	DLS	2	1.38	0.18	2.042	51.02	1.124

SCS: social and communication skills, ERS: emotion recognition and regulation skills, DLS: daily living skills, CS: cognitive skills, N: sample size, g: summary effect size estimate, SEg: standard error of g estimate, Q: heterogeneity statistic, I2: relative heterogeneity, tau2: between study variance

Table 5: meta-regression results of controlled trials

Moderator	Skill	N	Slope	p
number of sessions	**Overall**	45	-0.206	**0.0023**
	**SCS**	26	-0.268	**0.0046**
	ERS	14	-0.129	0.3232
gender	Overall	45	5.9204	0.5795
	**SCS**	26	-39.78	**0.0081**
	**ERS**	14	132.51	**0.0012**
publication date	Overall	45	-0.416	0.1737
	**SCS**	26	-1.022	**0.0187**
	**ERS**	14	-6.368	**0.0035**

SCS: social and communication skills, ERS: emotion recognition and regulation skills, N: number of estimates
